# A nanoemulsion targeting adipose hypertrophy and hyperplasia shows anti-obesity efficiency in female mice

**DOI:** 10.1038/s41467-023-44416-3

**Published:** 2024-01-02

**Authors:** Yichao Lu, Zhenyu Luo, Huanli Zhou, Yingying Shi, Ying Zhu, Xuemeng Guo, Jiaxin Huang, Junlei Zhang, Xu Liu, Sijie Wang, Xinyu Shan, Hang Yin, Yongzhong Du, Qingpo Li, Jian You, Lihua Luo

**Affiliations:** 1 College of Pharmaceutical Sciences, Zhejiang University, 866 Yuhangtang Road, Hangzhou, Zhejiang 310058 PR China; 2 State Key Laboratory for Diagnosis and Treatment of Infectious Diseases, 79 Qingchun Road, Shangcheng District, Hangzhou, Zhejiang 310006 PR China; 3 The First Affiliated Hospital, College of Medicine, Zhejiang University, 79 QingChun Road, Hangzhou, Zhejiang 310000 PR China; 4 Jinhua Institute of Zhejiang University, 498 Yiwu Street, Jinhua, Zhejiang 321299 PR China

**Keywords:** Diseases, Drug discovery, Risk factors

## Abstract

Obesity often leads to severe medical complications. However, existing FDA-approved medications to combat obesity have limited effectiveness in reducing adiposity and often cause side effects. These medications primarily act on the central nervous system or disrupt fat absorption through the gastrointestinal tract. Adipose tissue enlargement involves adipose hyperplasia and hypertrophy, both of which correlate with increased reactive oxygen species (ROS) and hyperactivated X-box binding protein 1 (XBP1) in (pre)adipocytes. In this study, we demonstrate that KT-NE, a nanoemulsion loaded with the XBP1 inhibitor KIRA6 and α-Tocopherol, simultaneously alleviates aberrant endoplasmic reticulum stress and oxidative stress in (pre)adipocytes. As a result, KT-NE significantly inhibits abnormal adipogenic differentiation, reduces lipid droplet accumulation, restricts lipid droplet transfer, impedes obesity progression, and lowers the risk of obesity-associated non-alcoholic fatty liver disease in female mice with obesity. Furthermore, diverse administration routes of KT-NE impact its in vivo biodistribution and contribute to localized and/or systemic anti-obesity effectiveness.

## Abstract (web summary)

Adipose tissue enlargement involves adipose hyperplasia and hypertrophy, which correlate with excessive ROS and hyperactivated XBP1. Here, the authors introduce KT-NE, a nanoemulsion combining KIRA6 (an XBP1 inhibitor) and α-Tocopherol, easing ER and oxidative stress in (pre)adipocytes and showing anti-obesity effectiveness.

## Introduction

With an estimated over 600 million people worldwide with obesity^1^, this chronic and debilitating metabolic disease is characterized by excessive adipogenesis and the accumulation of adipose tissue. It significantly increases the risk of multiple obesity-related complications, including but not limited to hepatic steatosis^2^, insulin resistance^3^, type 2 diabetes^4^, cancer^5,6^ and cardiovascular diseases^7,8^. Given that obesity is theoretically preventable and controllable, effectively managing body weight and fat becomes crucial for maintaining organismal fitness and homeostasis.

Despite the high prevalence of severe obesity worldwide and the associated health-devastating comorbidities, safe and efficient pharmacological interventions to combat obesity remain elusive^9–12^. Currently, there are only six major FDA-approved drugs for systemic anti-obesity use: orlistat, lorcaserin, phentermine, phentermine/topiramate extended release, naltrexone/bupropion sustained release and liraglutide (the only injectable preparation)^9^. Notably, among these medications, only orlistat acts as a reversible inhibitor of gastric and pancreatic lipases to restrict fat absorption^13^. However, it may cause progressive oxalate-induced acute kidney injury and various gastrointestinal (GI) adverse effects^14,15^. Additionally, other drugs mainly act through the central nervous system (CNS) pathways to either increase satiety or decrease appetite, correspondingly, potentially resulting in stimulant- or depressant-like nervous system-related adverse effects and neuroleptic malignant-like syndrome^9,16^. On the other hand, deoxycholic acid (Kybella™; Belkyra™) is currently the only approved treatment for local fat melt/lysis^17^. However, it may disrupt or lyse all cell membranes, including adipocytes, leading to considerable tissue necrosis and fibrosis of adipose and vascular tissues after accumulatively subcutaneous (s.c.) administration^18,19^. Consequently, no FDA-approved medications efficiently and safely achieve systemic anti-obesity effects along with local fat reduction efficacy in a heterogeneous population with obesity. A new therapeutic strategy with satisfactory pharmacological safety and robust anti-fat efficacy is urgently needed.

Adipogenesis in vivo is a complex physiological process occurring when aberrant enlargement of adipose tissue exists under the skin (s.c.) and around internal organs (visceral), leading to morbid corpulence. Expansion of s.c. adipose tissue (SAT) and visceral fat depots (VFD) primarly involves hypertrophy of existing adipocytes and adipocyte hyperplasia from committed preadipocytes^20^, including de novo lipogenesis and excessive lipid accumulation in (pre)adipocytes, physiologically correlating with oxidative stress triggered by overwhelming reactive oxygen species (ROS)^21,22^ along with overactivated inositol-requiring kinase 1α (IRE1α)-X-box binding protein 1 (XBP1), an endoplasmic reticulum (ER) stress sensor^23–25^. Previous research has shown that IRE1α-XBP1 hyperactivation occurs diffusely in the fatty tissue of humans with obesity^26^, and XBP1 splice is sequentially weakened during weight loss^27^.

**Fig. 1:**
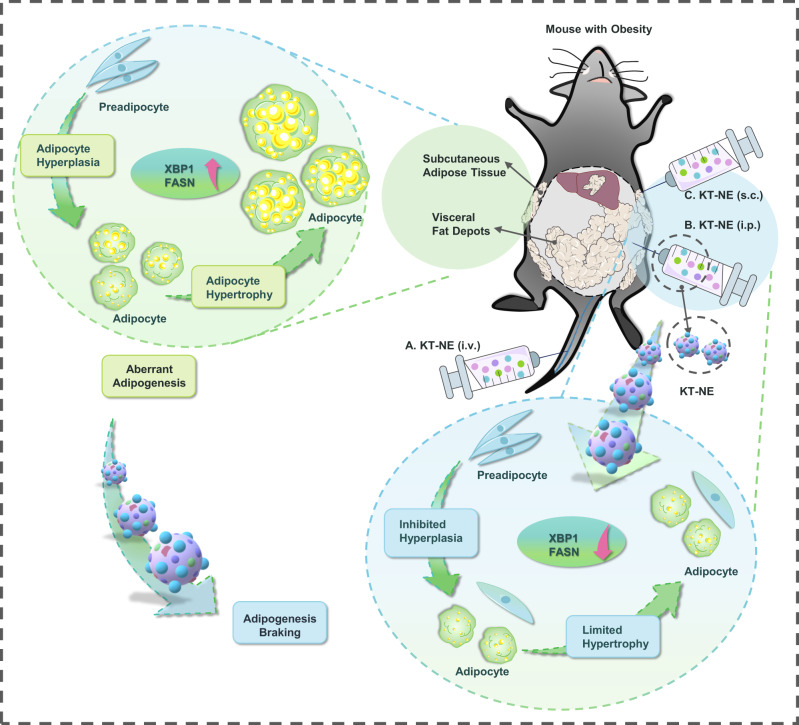
We here developed a nano-platform named KT-NE with the capability to alleviate endoplasmic reticulum stress and oxidative stress. KT-NE was formulated with the ROS scavenger α-Tocopherol and the XBP1 hyperactivation inhibitor KIRA6. This innovative platform impeded the advancement of obesity and mitigated the associated risk of complications by restricting uncontrolled adipose hyperplasia and hypertrophy in individuals with obesity.

We hypothesized that hyperactivated XBP1 and intracellular ROS play a fundamental role in orchestrating adipogenesis both in vivo and ex vivo, affecting adipogenic differentiation and adipose cells maturation^23^. Therefore, we aimed to construct a biocompatible stress-remission nanoemulsion to co-inhibit XBP1 overactivation and excessive ROS by employing KIRA6 loaded α-Tocopherol nanoemulsion (KT-NE). This nanoemulsion effectively restricted dysfunctional adipose tissue expansion, subsequently enhancing anti-obesity efficiency by inhibiting adipose hyperplasia and hypertrophy. Mechanistically, KIRA6, acts as an advanced small-molecule kinase and RNase inhibitor of IRE1α, the upstream activator of XBP1. It inhibits IRE1α-oligomerization-triggered cleavage of transcription factor XBP1 mRNA and its downstream signal transduction^28,29^. Simultaneously, α-Tocopherol exhibits strong biological activity to scavenge ROS, thereby relieving oxidative stress^30^. Our study sheds light on the link between XBP1 hyperactivation and adipogenesis, providing potential insights for designing safe and effective anti-obesity therapeutics for preclinical investigations and clinical applications (Fig. 1).

## Results

### XBP1 hyperactivation triggers adipose hyperplasia and hypertrophy

**Fig. 2:**
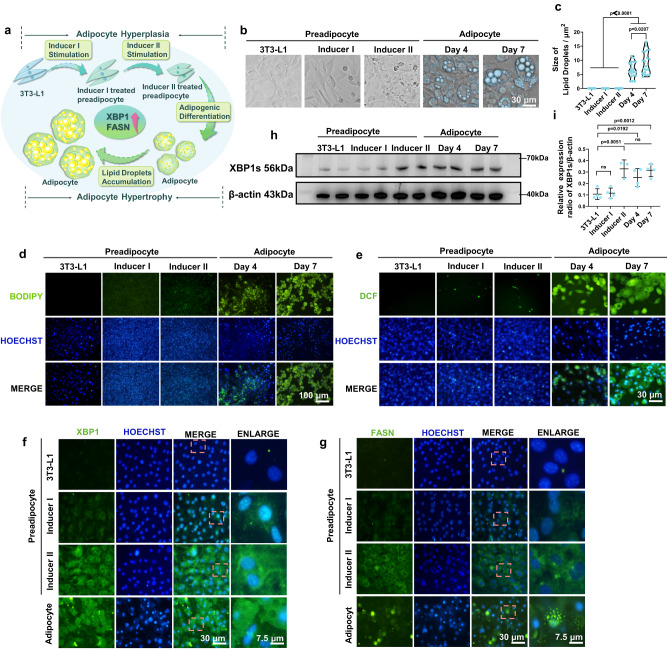
The excessive content of ROS content and hyperactivation of XBP1 drive adipogenesis. **a** The schematic diagram illustrating the pivotal role of XBP1s and its downstream factor FASN during adipogenic differentiation and adipose maturation. Two distinct groups of 3T3-L1 preadipocytes were described: those stimulated solely by Inducer I were referred to as Inducer I treated preadipocytes, and those successively stimulated by Inducer I and Inducer II were termed Inducer II treated preadipocytes. **b** Changes in cellular morphology and (**c**) intracellular lipid droplet size during (pre)adipocyte adipogenic development, with blue spheres representing intracellular lipid droplets. The time points after Inducer II treatment are labeled as follows: 2 days after treatment as “day 0,” 6 days as “day 4,” and 9 days as “day 7” accordingly. Scale bar = 30 μm. *n* = 20 lipid droplets examined over three independent experiments. **d** Representative fluorescence images reflecting intracellular lipid content (scale bar = 100 μm) and (**e**) ROS level in (pre)adipocytes (scale bar = 30 μm). DCF Dichlorofluorescein. **f** Representative immunofluorescence images of XBP1s and (**g**) FASN expression in indicated (pre)adipocytes. Scale bar = 30 μm or 7.5 μm, respectively. **h** Western blot analysis (**i**) and quantification (*n* = 3 in the “Induce II” group and *n* = 4 in other groups) of XBP1s expression from indicated groups. All experiments were repeated three times independently, with similar results. Source data are provided as a Source Data file. All data was expressed as mean ± SD, ns no significance. Statistical significance was evaluated by an unpaired two-tailed t-test.

As prior studies have established, the massive augmentation of adipose tissue can be provoked either by the incremental number of adipocytes (hyperplasia through committed preadipocyte differentiation) or by expanded adipocyte size (hypertrophy through adipocyte lipid droplet accumulation), which closely entails the hyperactivated XBP1 and subsequent adipogenesis pathways^23,24,31–33^ (Fig. 2a).

To appraise whether XBP1 manipulates adipose hyperplasia and hypertrophy by serving as a pivotal transcriptional regulator during adipogenesis, the primary preadipocyte cell line 3T3-L1 was employed, which can undergo adipogenic differentiation after being stimulated by Inducer I and Inducer II in succession^34^. Preadipocytes, such as 3T3-L1, Inducer I treated preadipocyte and Inducer II treated preadipocyte, underwent significant morphological changes during the final stages of adipogenic differentiation. This process led to the substantial production, enlargement, and accumulation of abundant lipid droplets in adipocytes, following a time-dependent pattern throughout adipose maturation. (Fig. 2b–d). Moreover, in the course of adipose hyperplasia and hypertrophy, (pre)adipocytes exhibited elevated intracellular ROS level (Fig. 2e), whose content could mutually interact with XBP1 hyperactivation (or ER stress), as previous evidence^25,32,33^. Additionally, we observed that preadipocytes subjected to adipogenic stimulus and adipocytes in the maturation phase overexpressed XBP1s along with the downstream fatty acid synthetase (FASN), which is responsible for synthesizing fatty acids^35^ (Fig. 2f–i). This coincided with a robust upregulation in lipid droplet and ROS accumulation.

Taking into account the crucial role of XBP1 in promoting adiposity-related hyperplasia and hypertrophy, an effective strategy would involve inhibiting XBP1 hyperactivation. This inhibition could be achieved by alleviating ER and oxidative stress in (pre)adipocytes, thereby enhancing the effectiveness of anti-obesity treatments.

### Formulation and characterization of KIRA6-incorporated α-Tocopherol nanoemulsion

**Fig. 3:**
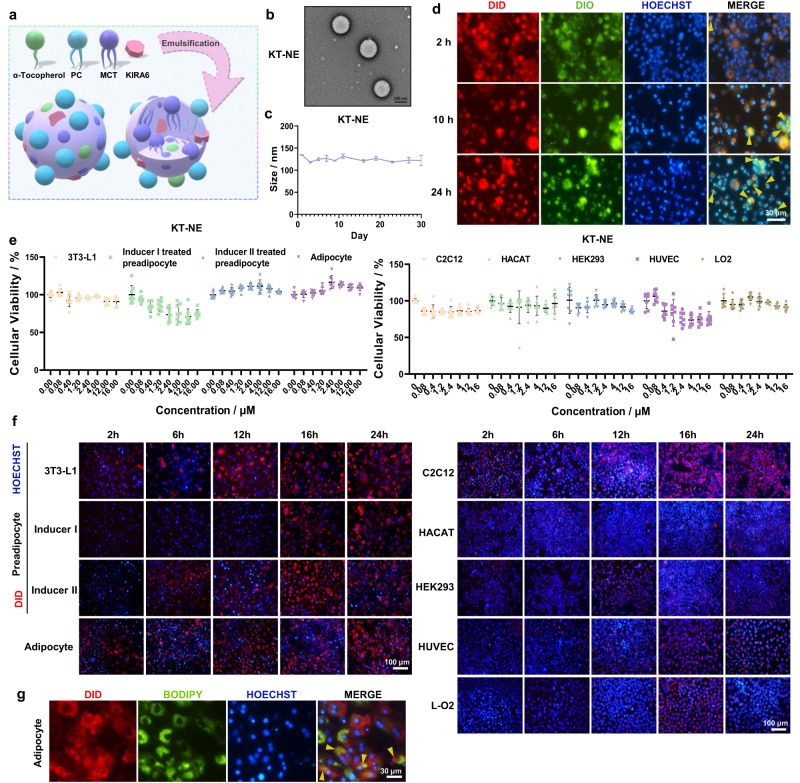
Construction and characterization of KT-NE. **a** Schematic illustration showing the ingredients and three-dimensional architecture of KT-NE. **b** Transmission electron microscope (TEM) images showcasing the morphologies of KT-NE. Scale bar = 100 nm. **c** Analysis of the particle diameter changes of KT-NE stored at 4 °C over a 30-day period. **d** Temporal profiling of drug release kinetics involving DID- (red) and DIO- (green) labeled KT-NE within adipocytes over a 24-h interval. Noteworthy, the yellow triangles denote instances of representative non-colocalization within adipocytes, indicative of the drug release behavior. Scale bar=30 μm. **e** Dose-dependent cell viability of various cells (3T3-L1 preadipocytes, Inducer I treated preadipocytes, Inducer II treated preadipocytes, adipocytes, C2C12, HACAT, HEK-293, HUVEC, and L-O2) subsequent to 24-h incubation with KT-NE at varying drug concentrations. *n* = 8. Statistical significance was evaluated by an unpaired two-tailed t-test. **f** Time-dependent cellular uptake of DID-labeled KT-NE by (pre)adipocyte subgroups (3T3-L1 preadipocytes, Inducer I treated preadipocytes, Inducer II treated preadipocytes, and adipocytes) and normal somatic cell lines (C2C12, HACAT, HEK-293, HUVEC, and L-O2). Representative fluorescent micrographs of cellular uptake were systematically captured at 2 h, 6 h, 12 h, 16 h, and 24 h, respectively. Scale bar = 100 μm. **g** Representative fluorescent images depicting partial co-location of DID-labeled KT-NE and lipid droplets within adipocytes following a 24-h incubation. Yellow triangles indicate representative co-location or partial co-location of DID-labeled nanoemulsions and BODIPY-dyed lipid droplets within adipocytes. Scale bar = 30 μm. All experiments were repeated three times independently, with similar results. Source data are provided as a Source Data file. All data was expressed as mean ± SD.

Considering the fact that elevated ROS levels and XBP1 hyperactivation positively correlate with adipose tissue development, a nanoemulsion (KT-NE) was formulated. This is comprised of phosphatidylcholine (PC), medium chain triglyceride (MCT), anti-oxygen component α-Tocopherol, and KIRA6 (inhibitor of XBP1 mRNA cleavage) and underwent emulsification and probe-sonication (Fig. 3a). Similarly, three other nanoscale emulsions (Bank-NE, T-NE, and K-NE) were fabricated analogously (Supplementary Fig. 1a).

Exhamination through transmission electron microscope revealed that all NEs were characterized by a typical spherical structure with a diameter of 100–150 nm (Fig. 3b, Supplementary Fig. 1b). These structures remained stable for at least 1 month at 4 °C (Fig. 3c, Supplementary Fig. 1c). Additionally, to assess the intracellular drug release attributes of KT-NE, oil-soluble dyes DID (red), and DIO (green) were utilized to mimic KIRA6 and α-Tocopherol. In instances where the dyes did not completely co-localize in adipocytes, it indicated that drug release had occurred. Notably, most drugs remained encapsulated inside the carrier even after 2 h, with gradual release observed between 10 and 24 h (Fig. 3d).

In light of safety concerns surrounding anti-obesity and fat-melt/lysis treatments in people with obesity, we next evaluated the cytotoxicity over 24 h of four preparations across (pre)adipocyte subgroups (3T3-L1 preadipocytes, Inducer I treated preadipocytes, Inducer II treated preadipocytes and adipocytes) and normal somatic cell lines (C2C12, HACAT, HEK-293, HUVEC and L-O2). These formulations all had negligible toxicity up to 16 μM for 24 h in both (pre)adipocyte subpopulations and normal somatic cells, maintaining over 80% cell (Fig. 3e, Supplementary Fig. 1e). Moreover, when pre-labeled nanoscale emulsions were used to compare cellular uptake capacity within 24 h, the (pre)adipocytes demonstrated superior capacity for internalization and phagocytosis compared to normal somatic cells (Fig. 3f), which may be attributed to their inherent affinity for lipid-like nanoscale emulsions (Fig. 3g).

Arguably, these low-toxicity nanoscale emulsions might effectively target (pre)adipocytes passively after s.c. or systemic administration, potentially reducing adverse effects in vivo.

### KT-NE inhibits adipogenesis

**Fig. 4:**
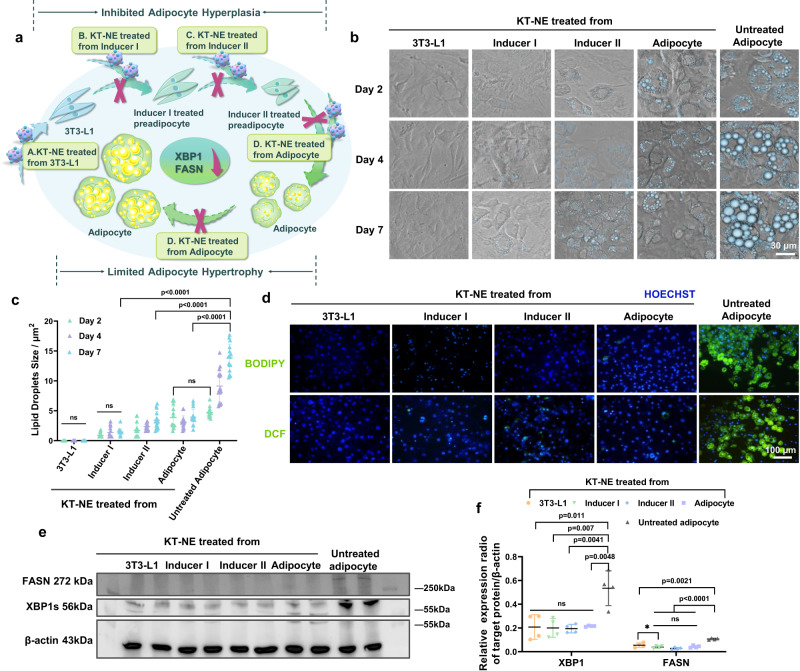
KT-NE possesses anti-adipogenesis effectiveness. **a** Schematic representation delineating the protocol for KT-NE treatment. (Pre)adipocytes underwent culture with KT-NE at various stages of differentiation or maturation, with the study concluding on day 7 (9 days after Inducer II treatment). For instance, if KT-NE treatment commenced at the 3T3-L1 stage, it implies continuous administration throughout Inducer I and Inducer II stimulation until the culmination of the experiment (day 7). **b** Evolution of cellular morphology and (**c**) alterations in intracellular lipid droplet size observed in specified groups (subject to KT-NE treatment initiation at distinct time points) on day 2, day 4, and day 7. The blue spheres denote lipid droplets. Scale bar = 30 μm. **d** Representative fluorescence images depicting intracellular lipid content and ROS levels in designated groups at day 7. Scale bar = 100 μm. **e** Western blot analysis and (**f**) corresponding quantification of XBP1s and FASN expression in the indicated groups. *n* = 4. Statistical significance was evaluated by an unpaired two-tailed t-test. All experiments were repeated three times independently, with similar results. Source data are provided as a Source Data file. All data was expressed as mean ± SD, ns no significance.

As researchers have already reported, the remission of either ER stress or oxidative stress significantly contributes to adipogenesis^24,33,36,37^. Thus, we attempted to investigate whether ER-stress- and oxidative-stress-remission nanoplatforms KT-NE exert a potent influence on limiting adipose hyperplasia and hypertrophy. Consequently, we incubated KT-NE with (pre)adipocytes in various phases of differentiation or maturation but terminated the study at same phase (Fig. 4a). To elaborate, the initial KT-NE treatment began with the different differentiation phase of preadipocytes—namely, 3T3-L1 preadipocytes, Inducer I treated preadipocytes, or Inducer II treated preadipocytes—or with differentiated adipocytes, respectively. The application of KT-NE in various preadipocytes or adipocytes persisted throughout the entire period until day 7 post the completion of adipogenic differentiation. Notably, here, the term “day 0” referred to the time point 2 days after Inducer II treatment, while “day 7” corresponded to 9 days after Inducer II treatment. It’s noteworthy that all preadipocytes underwent the same Inducer I and Inducer II stimulation throughout the entire course of KT-NE treatment.

Impressively, we discovered that KT-NE significantly restrained the development of both the number and the size of adipocytes compared with the untreated control, regardless of when KT-NE treatment started or how long it lasted (Fig. 4b, c). When initial KT-NE treatment began with naive 3T3-L1 preadipocytes, a striking observation was the scarcity of rounded differentiated adipocytes, contrasting with the abundant presence of fusiform undifferentiated preadipocytes. Then, we estimated the intracellular lipid droplet and ROS content from the indicated groups at “day 7” and KT-NE treated groups displayed a significant decrease in lipid droplet and ROS intracellular storage (Fig. 4d), which may be linked with the downregulation of XBP1s and FASN expression (Fig. 4e, f).

In brief, KT-NE contributed to restricting adipose hyperplasia and hypertrophy, especially adipose hyperplasia. Notably, the earlier the (pre)adipocytes were co-cultured with KT-NE, the more effective the anti-adipogenesis outcomes were obtained.

### Co-relieving ER stress and oxidation stress enhances anti-adipogenesis efficiency

Encouraged by the anti-adipogenesis effect, particularly the blockade of adipose hyperplasia by KT-NE (Fig. 4b–d), we aimed to focus on the restriction of adipose hypertrophy by KT-NE. We sought to ascertain whether co-remission of ER stress and oxidation stress could enhance the anti-adipogenesis effectiveness in adipocytes (at “day 7”) via treatment with NEs (T-NE, K-NE, KT-NE) for 72 h.

**Fig. 5:**
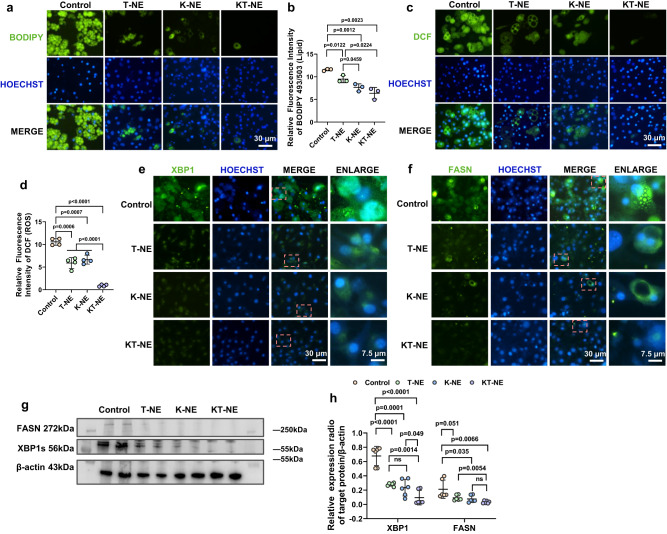
Synergistic ER stress and oxidative stress inhibition mitigates adipose hypertrophy. **a** Representative fluorescence micrographs and (**b**) quantification of intracellular lipid droplet storage in adipocytes post Nanoemulsion treatment (T-NE, K-NE, KT-NE) on day 7 (*n*  =  3). Statistical significance was evaluated by an unpaired two-tailed t-test. Scale bar = 30 μm. **c** Representative fluorescence images and (**d**) corresponding quantification of intracellular ROS levels in adipocytes following treatment with nanoemulsions (T-NE, K-NE, KT-NE) on day 7 (*n*  =  4). Statistical significance was evaluated by an unpaired two-tailed t-test. Scale bar = 30 μm. **e**, **f** Representative immunofluorescence images and (**g**) representative western blot analyses, with (**h**) corresponding quantification of XBP1s and FASN expression in adipocytes treated with T-NE, K-NE, KT-NE on day 7. Scale bar = 30 μm, and scale bar = 7.5 μm in enlarged images. *n* = 6. Statistical significance was evaluated by an unpaired two-tailed t-test. All experiments were repeated three times independently, with similar results. Source data are provided as a Source Data file. All data was expressed as mean ± SD, ns no significance.

Impressively, KT-NE, the ER stress- and oxidation stress- co-relieving nano-platform, led to a remarkable reduction in lipid accumulation in adipocytes (Fig. 5a, b), which coincided with a sharp decrease in intracellular ROS storage (Fig. 5c, d). In line with this, KT-NE-treated adipocytes displayed a prominent downregulation of XBP1s and FASN expression (Fig. 5e–h), especially compared to the untreated control. Hence, KT-NE exhibited a notable inhibition of adipose hypertrophy, hindering the expansion of adipocyte size mainly dependent on intracellular lipid droplet accumulation. Simultaneously, the co-inhibiting nanosystem targeting ER stress and oxidation stress limited adipogenesis driven by hyperactivated XBP1 and excessive ROS.

### KT-NE limits lipid droplets transfer among adipocytes

The progression of obesity is a complex that unfolds like a cascade, with adipocytes actively engaged^38^. In essence, within the progression of excessive adipose hypertrophy, adipocytes have the capacity to promote adipose hyperplasia and hypertrophy in dormant preadipocytes and adipocytes by transmitting adipogenic signals. These adipogenic signals may include: (1) direct delivery of lipid droplets to directly enlarge inherent lipid droplets in recipient adipocytes; (2) adipogenesis-boosted adipocytokines to indirectly activate lipid droplets biosynthesis program in recipient adipocytes^38^. Moreover, as we have shown that these (pre)adipocytes, upon KT-NE internalization, could block adipogenic differentiation and lipid droplets accumulation by downregulating XBP1s and FASN (Fig. 4f). Therefore, we sought to determine whether these adipocytes with endocytosed or internalized KE-NE could impede adipose progression in the distal adipocytes that did not undergo KT-NE internalization. Thus, the sequential disruption in the cascaded process of adipose hyperplasia and hypertrophy may occur through the mechanism described above.

**Fig. 6:**
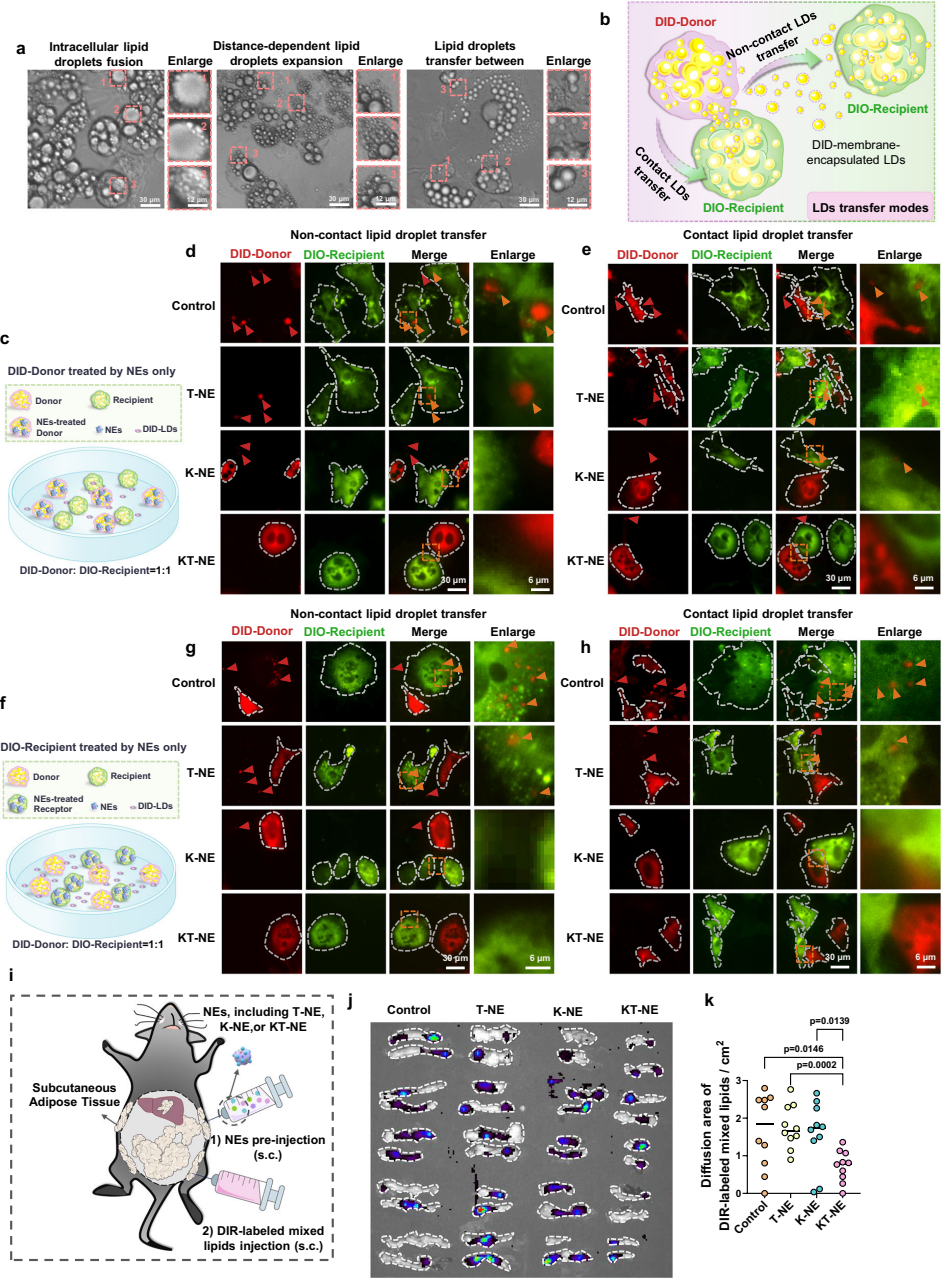
KT-NE restricts intercommunication among adipocytes in vitro and in vivo. **a** Representative digital images depicting the dynamics of lipid droplets (LDs). Experiments were repeated three times independently, with similar results. Scale bar = 30 μm, and scale bar = 12 μm in enlarged images. **b** Schematic overview illustrating contact-dependent and contact-independent LDs transfer between DID-labeled donor adipocytes and DIO-marked recipient adipocytes. **c** Schematic outline detailing the co-culturing of KT-NE-treated DID-labeled donor adipocytes (red) and untreated DIO-labeled recipient adipocytes (green). **d** Representative fluorescence images demonstrating non-contact-dependent LDs transfer and (**e**) contact-dependent LDs transfer between DID-labeled donor adipocytes (KT-NE treated, red) and DIO-marked recipient (untreated) adipocytes (green). Extracellular/intracellular red dots represent DID-marked membrane-encapsulated small lipid droplets secreted by DID-marked donor adipocytes (red), highlighted with red triangles. Internalized and accumulated DID-marked membrane-encapsulated small lipid droplets into DIO-recipient (especially into the larger lipid droplets) were marked with orange triangles. Experiments were repeated three times independently, with similar results. Scale bar = 30 μm, and scale bar = 6 μm in enlarged images. **f** Diagrammatic sketch of co-culturing untreated DID-labeled donor adipocytes (red) and KT-NE-treated DIO-labeled recipient adipocytes (green). **g** Representative fluorescence images demonstrating non-contact-dependent LDs transfer and (**h**) contact-dependent LDs transfer between DID-labeled donor adipocytes (untreated, red) and DIO-marked recipient (KT-NE treated) adipocytes (green). Experiments were repeated three times independently, with similar results. Scale bar = 30 μm, and scale bar = 6 μm in enlarged images. **i** Sketch map illustrating DIR-labeled mixed lipids biodistribution in mice with obesity (*n* = 5 biologically independent animals) pre-treated with indicated NEs for 1 week. **j** Biodistribution and (**k**) relative fluorescence transmission (or diffusion) distance/area of DIR-labeled mixed lipids in subcutaneous adipose tissue (SAT), after DIR-labeled mixed lipids injected (s.c.) into the hosts for 48 h. *n* = 10 SAT from five biologically independent animals. Source data are provided as a Source Data file. Statistical significance was evaluated by an unpaired two-tailed t-test. All data was expressed as mean ± SD, ns no significance.

We found that the newly formed droplets were recruited and fused into larger ones, with the size of lipid droplets exhibiting a distance-dependent trend, indicating an increase in droplet size closer to the cell’s center (Fig. 6a). Furthermore, some adipocytes appeared to transfer lipid droplets among connected adipocytes (Fig. 6a). In line with previous literatures, all these findings further corroborated that lipid droplets were highly dynamic and could enlarge based on intercommunication among adipocytes^38,39^. Notably, though the mechanisms of adipocyte interaction remain enigmatic, the communication between cells with active membrane processes involves complex mediation through contact-dependent pathways (e.g., tunneling nanotubes and gap junctions) as well as contact-independent ways (e.g., extracellular vesicles)^40–43^. Therefore, we next attempted to explore lipid droplets transfer modes among adipocytes and hypothesized that such transfer could occur via contact-based ways or non-contact-based ways (Fig. 6b).

Subsequently, we conducted experiments where adipocytes pre-labeled with membrane-visualized fluorescent dyes DID (red) acted as the droplet donors, and adipocytes pre-labeled with dyes DIO (green) served as the recipients. After co-culturing the donor and recipient adipocytes for 24 h, we observed that the holes in membrane-visualized dye-labeled adipocytes corresponded to the locations of their intracellular lipid droplets (Supplementary Fig. 2a). Intriguingly, the membrane-encapsulated small lipid droplets labeled with DID from the donor were precisely delivered into the holes (intrinsic lipid droplets) of the DIO-labeled recipient either through contact-based or non-contact-based means (Supplementary Fig. 2b). Strikingly, after DID-labeled donor and/or DIO-labeled recipient were treated with KT-NE, the transfer of lipid droplets, whether through contact-based or non-contact-based pathways, was restrained (Fig. 6c–h, Supplementary Fig. 2c–e). This observed effect may be closely related to the fact that inhibited XBP1 superactivation led to reduced membrane fluidity and lipids uptake capability^44,45^. Therefore, KT-NE might effectively impede the vigorous expansion of adipocytes, interrupting the cascaded transmission and intercommunication of adipogenic signals. This restrained transfer of lipid droplets among adipocytes could significantly contribute to limiting the domino-style progression of adipose hyperplasia and hypertrophy.

Encouraged by the effective LDs transfer blockade triggered by KT-NE ex vivo, we proceeded to examine whether KT-NE could impede lipids absorption and transmission (or diffusion) abilities in adipose tissues. Female mice with high-fat-diet (HFD)-induced obesity, pre-treated with multi-point administration (s.c.) of total two doses of NEs (T-NE, K-NE or KT-NE) in 1 week, were injected with DIR-labeled mixed lipids solution (s.c., multi-point), and they were sacrificed after 48 h to assess lipids transmission and diffusion in adipose tissue (Fig. 6i). Mixed lipids were predominantly confined to the injection sites with minimal transmission and diffusion in the adipose tissues of mice pre-treated with KT-NE compared to mice pre-treated with other NEs (Fig. 6j–k, Supplementary Fig. 3a, b). This outcome suggests that KT-NE could significantly restrict lipids absorption, transmission, and diffusion in adipose tissue in vivo.

Generally, the augmentation of adipocytes in the deeper layers of adipose tissue relies heavily on the transmission of adipogenic signals from adipocytes closed to blood vessels due to the difficulty in long distance free FAs transportation^38^. This situation further accentuates the domino-like cascade of adipose development within fatty tissues. Consequently, delaying or directly inhibiting lipid transmission in fat depots using KT-NE could prove to be an effective strategy in combating obesity.

### KT-NE induces anti-obesity effect

**Fig. 7:**
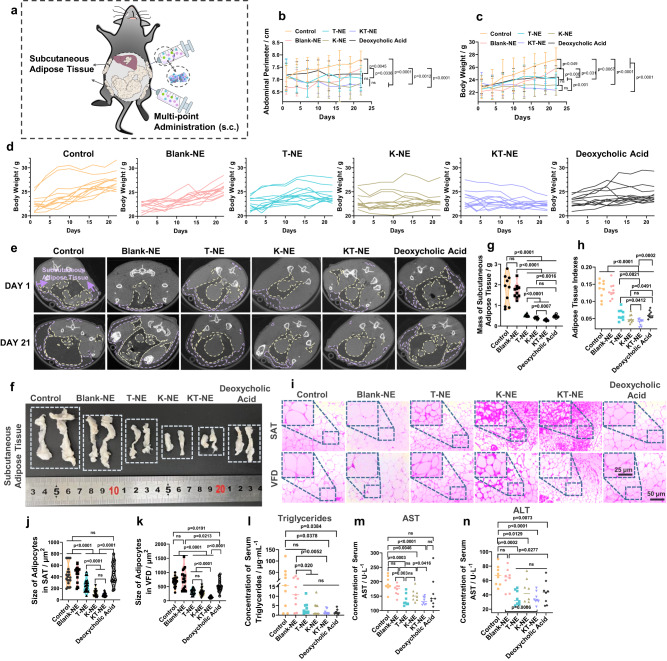
Subcutaneous administration of KT-NE elicits anti-obesity effects. **a** Schematic representation of therapeutic interventions in a high-fat diet (HFD)-induced obesity model in female C57BL/6 mice. Following the establishment of the HFD-induced obesity model, the mice with obesity received a total of six subcutaneous doses of various formulations over 3 weeks (multi-point). Body weight gain and abdominal perimeter were monitored until day 22 (*n* = 12 biologically independent animals in each group). **b** Abdominal perimeter curves and (**c**, **d**) body weight growth curve for mice receiving different formulations. (*n* = 12 biologically independent animals). Statistical significance was evaluated by an unpaired two-tailed t-test. **e** Micro-CT results depicting changes in body fat distribution in mice with obesity on day 1 and day 21. Darker areas indicate regions of lower density, with subcutaneous adipose tissue delineated by the purple dotted line frame and visceral fat depots by the yellow dotted line frame. **f** Representative digital image and (**g**) corresponding quantification of bilateral inguinal subcutaneous adipose tissue from indicated groups. (*n* = 10 biologically independent animals). Statistical significance was evaluated by an unpaired two-tailed t-test. **h** Quantification of adipose tissue indexes (total mass of adipose tissue/body weight, and total mass of adipose tissue = subcutaneous adipose tissue + visceral fat depots) for indicated groups. (*n* = 9 biologically independent animals). Statistical significance was evaluated by an unpaired two-tailed t-test. **i** Representative H&E staining images and (**j**) quantification of adipocyte size in subcutaneous adipose tissue and (**k**) visceral fat depots. Scale bar = 50 μm, and scale bar = 25 μm in enlarged images. *n* = 20 cells examined over three independent experiments. Statistical significance was evaluated by an unpaired two-tailed t-test. **l** Serum triglycerides, (**m**) aspartate aminotransferase (AST), and (**n**) alanine aminotransferase (ALT) levels detected by commercial reagent kits (*n* = 8 biologically independent samples). Source data are provided as a Source Data file. Statistical significance was evaluated by an unpaired two-tailed t-test. All data was expressed as mean ± SD, ns no significance.

Considering KT-NE’s potential in promoting anti-adipogenesis ex vivo and its links to ROS and ER-stress signaling in HFD-induced adiposity^26,27,36,37^, we next sought to explore whether KT-NE could effectively elicit localized and systemic anti-adiposity in vivo. Given that C57BL/6 mouse strain has increased propensity to model metabolic perturbations and complications (e.g., insulin resistance and non-alcohol fatty liver disease) associated with obesity^33^, HFD-based C57BL/6 mice model with obesity was employed, which was established via an ad libitum HFD for 4 weeks (Supplementary Fig. 4a), resulting in significantly increased body weight, abdominal perimeter, and body fat (both in s.c. and visceral fat deposits) (Supplementary Fig. 4b–e). These mice received multi-point administration (s.c.) near bilateral inguinal SAT with indicated formulations (including PBS, Blank-NE, T-NE, K-NE, KT-NE and FDA-approved 10% deoxycholic acid for local fat lysis) twice-per-week, while the mice continued the HFD regimen throughout the treatment period (Fig. 7a). KT-NE showed high biocompatibility and biosafety (Supplementary Fig. 5a, b) and superior localized and systemic anti-adiposity efficacy compared with the other formulations, including FDA-approved therapeutic. This was evident in the thinner physique (Supplementary Fig. 5c), decreased abdominal perimeter (Fig. 7b), and body weight growth curve (Fig. 7c, d). Furthermore, the treatment reduced fat content in SAT (Fig. 7e), decreased fatty tissue mass (Fig. 7f, g, Supplementary Fig. 5d, e) and corresponding adipose tissue indexes (Fig. 7h). These outcomes may be related to the decreased ROS content and reduced XBP1s protein expression in adipose tissues of the HFD-fed mice (Supplementary Fig. 6a–f, Supplementary Fig. 7a, b). Additionally, detailed tissue analysis further revealed that KT-NE-treated mice with obesity featured smaller adipocyte sizes in both SAT and VFD (Fig. 7i–k), indicating KT-NE s.c. injection limited adipose hypertrophy in vivo, supporting its anti-obesity effect.

Previous studies have shown that non-alcoholic fatty liver disease (NAFLD) is burden in obesity, potentially leading to severe conditions such as cirrhosis, liver failure, and hepatocellular carcinoma ultimately^46^. NAFLD, which emerged as the most common indication for liver transplantation^47^, is characterized by hepatic steatosis, dyslipidemia, and impaired biological function. Serum concentrations of alanine aminotransferase (ALT) and aspartate aminotransferase (AST) are regarded as biomarkers for noninvasive assessment of NAFLD in clinical diagnosis and epidemiological researches^47,48^. In these regards, we used H&E staining to assess hepatic steatosis and commercial reagent kits to evaluate serum concentration of NAFLD biomarkers (triglycerides, AST, and ALT). KT-NE-treated mice with obesity had less hepatic steatosis (Supplementary Fig. 5a), as well as lower triglycerides, AST and ALT levels in serum (Fig. 7l–n). These changes may closely correlate with the reversal of obesity and downregulation of liver XBP1 expression (Supplementary Fig. 8a–d) in KT-NE-treated mice with obesity.

### Different administration routes influence KT-NE in vivo biodistribution

**Fig. 8:**
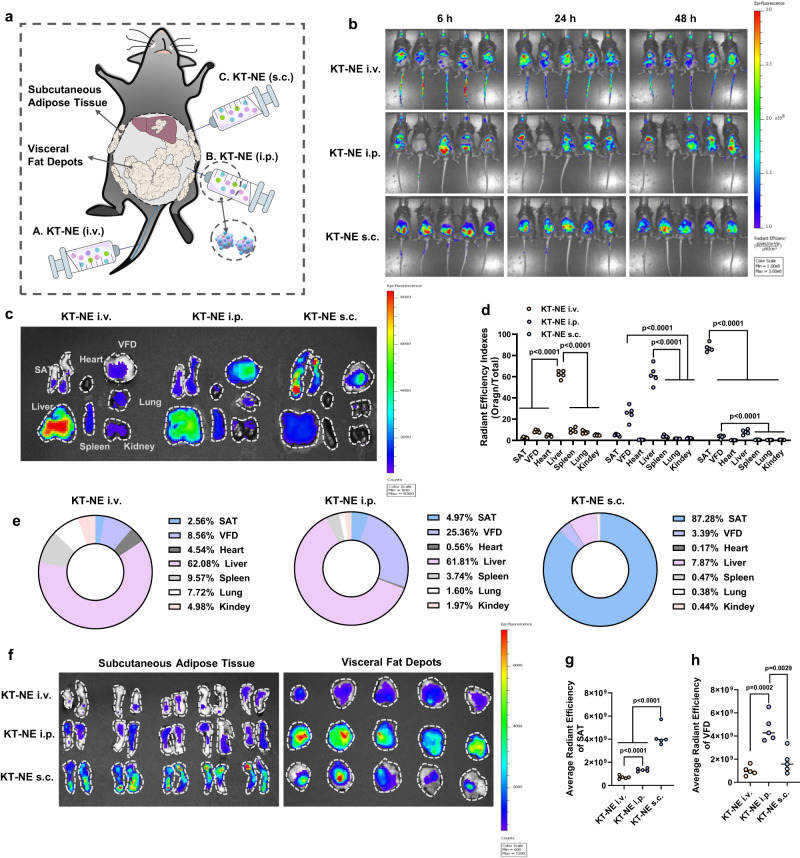
Delivery routes impact KT-NE biodistribution in vivo. **a** Schematic illustration of KT-NE administration routes (i.v., i.p., and s.c.). **b** Biodistribution of DIR-labeled KT-NE at 6 h, 24 h, and 48 h after indicated injection (*n* = 5 biologically independent mice). **c** Biodistribution and (**d**, **e**) relative fluorescence intensity efficiency indexes of DIR-labeled KT-NE in subcutaneous adipose tissue (SAT), visceral fat depots (VFD), heart, liver, spleen, lung and kidney of mice with obesity, after KT-NE injected (i.v., i.p., and s.c. respectively) into the hosts for 48 h. **f** Biodistribution and (**g**, **h**) relative fluorescence intensity efficiency indexes of DIR-labeled KT-NE in SAT (**g**) and VFD (**h**) of mice with obesity after KT-NE injected (i.v., i.p., and s.c. respectively) into the hosts for 48 h. *n* = 5 biologically independent animals. Source data are provided as a Source Data file. Statistical significance was evaluated by an unpaired two-tailed t-test. All data was expressed as mean ± SD.

Given that s.c. injected KT-NE showed greater efficacy in SAT near to the injection sites compared with VFD, we aimed to investigate how various administration routes could impact KT-NE biodistribution in vivo. To track KT-NE in vivo, we used an oil-soluble fluorescent dye DIR, which was administrated to HFD-fed mice with obesity through direct intravenous (i.v.), intraperitoneal (i.p.), and s.c. injections near bilateral inguinal SAT (Fig. 8a). We then evaluated the biodistribution in SAT, VFD, and the major visceral organs 48 h after administration.

The results indicated that s.c. injected KT-NE tended to remain at the injection sites within 48 h, whereas KT-NE injected via i.v. and i.p. showed systemic distribution (Fig. 8b). Specifically, s.c. administrated KT-NE mainly accumulated in SAT within 48 h post administration, with partial distribution in the liver and VFD (Fig. 8c–e, Supplementary Fig. 9). Interestingly, after i.p. injection, the majority of KT-NE accumulated in the liver and VFD, with minimal presence in SAT, and little accumulation in other visceral organs (Fig. 8c–e, Supplementary Fig. 9). On the contrary, most intravenously injected KT-NE was absorbed by the liver and lung, with a small amount present in adipose tissues (Fig. 8c–e, Supplementary Fig. 9). Subcutaneously injected KT-NE exhibited a superior ability to accumulate in SAT, while i.p. administration led to the highest accumulation in VFD, and i.v. administration resulted in the least accumulation in adipose tissues (Fig. 8f–h). Hence, diverse delivery routes of KT-NE exhibited different biodistribution, potentially leading to diverse localized and/or systemic anti-obesity outcomes.

### KT-NE delivered via different routes elicits diverse localized and systemic anti-adiposity effects

**Fig. 9:**
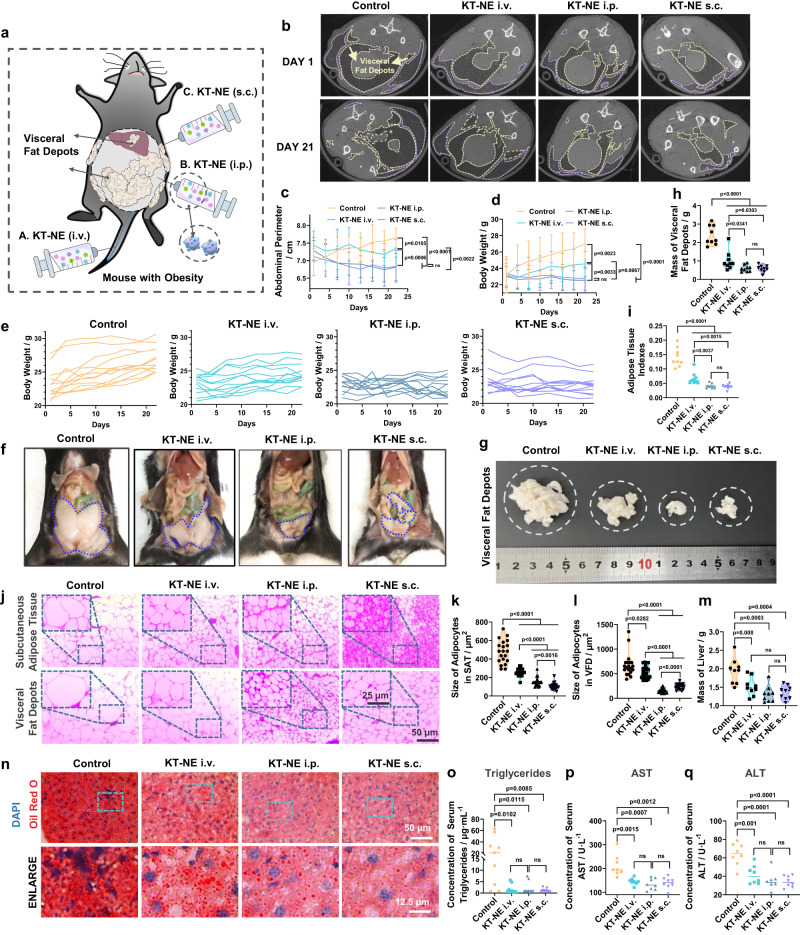
Administration routes affect localized and systemic anti-adiposity effects of KT-NE. **a** Schematic illustration delineating various injection strategies in high-fat diet (HFD)-induced mice with obesity. Following the establishment of the HFD-induced obesity model, the C57BL/6 female mice with obesity received six doses of KT-NE via various administration routes (i.v., i.p. and s.c.) over 3 weeks. Subsequent monitoring of body weight growth and abdominal perimeter extended until day 22 (*n* = 12 biologically independent animals in each group). **b** Micro-CT showcasing alterations in body fat distribution of mice with obesity on day 1 and day 21. Regions with lower density, symbolized by darker areas, elucidate subcutaneous adipose tissue (purple dotted line frame) and visceral fat depots (yellow dotted line frame). **c** Abdominal perimeter curve and (**d**, **e**) body weight growth curve for mice subjected to KT-NE treatment through i.v., i.p. or s.c. injection. (*n* = 12 biologically independent animals). Statistical significance was evaluated by an unpaired two-tailed t-test. **f**, **g** Representative digital image and (**h**) corresponding quantification of visceral fat depots from indicated groups (*n* = 9 biologically independent samples). Statistical significance was evaluated by an unpaired two-tailed t-test. **i** Quantification of adipose tissue indexes (total mass of adipose tissue/body weight, and total mass of adipose tissue = subcutaneous adipose tissue + visceral fat depots) of indicated groups (*n* = 9 biologically independent samples). Statistical significance was evaluated by an unpaired two-tailed t-test. **j** Representative H&E staining images and (**k**) quantification of adipocyte size of subcutaneous adipose tissue and (**l**) visceral fat depots. Scale bar=50 μm, and scale bar=25 μm in enlarged images. n = 20 cells examined over 3 independent experiments. Statistical significance was evaluated by an unpaired two-tailed t-test. **m** Quantification of liver mass of indicated groups (*n* = 9 biologically independent samples). Statistical significance was evaluated by an unpaired two-tailed t-test. **n** Representative liver Oil Red O staining images from indicated groups. Scale bar = 50 μm, and scale bar = 12.5 μm in enlarged images. **o** Serum triglycerides, (**p**) aspartate aminotransferase (AST), and (**q**) alanine aminotransferase (ALT) levels detected by commercial reagent kits (*n* = 8 biologically independent samples). Source data are provided as a Source Data file. Statistical significance was evaluated by an unpaired two-tailed t-test. All data was expressed as mean ± SD, ns no significance.

Encouraged by the positive anti-obesity results of KT-NE delivered s.c, especially for SAT (Fig. 7e), and given the role of ROS in diet-triggered visceral adiposity progression^37^, we investigated whether KT-NE delivered through diverse routes could lead to different localized and systemic anti-obesity effects. This also considering that various administration strategies strikingly influenced KT-NE biodistribution in adipose tissues and major organs (Fig. 8c–e). After establishing an obesity model in mice through a HFD (Supplementary Fig. 4a), these mice were randomly divided into groups and received the same dosage of KT-NE through different delivery methods including i.v. injection, i.p. injection, and s.c. multi-point administration, while maintaining the persistent HFD feeding regimen (Fig. 9a).

Notably, during the continuous high-calorie uptake period (Supplementary Fig. 10a), KT-NE delivered through systemic administration routes (i.v. and i.p.) or localized administration way (s.c.) showed a tendency to delay or inhibit adipose tissue expansion (Fig. 9b), control or reduce abdominal perimeter (Fig. 9c), body weight (Fig. 9d, e, Supplementary Fig. 10b), and reduce adipose tissue indexes (Fig. 9f–i, Supplementary Fig. 10c, d) in comparison to the untreated control. Although KT-NE delivered via different routes induced anti-adiposity effect to some extent, KT-NE (s.c) notably attenuated the enlargement of SAT (Supplementary Fig. 10c, d), and KT-NE (i.p) boosted a promoted efficacy in systemic anti-adiposity and limiting VFD enlargement (Fig. 9b–d, f). These effects may be linked to the decreased oxidative stress and ER stress in SAT and/or VFD (Supplementary Fig. 11a, Supplementary Fig. 11b, Supplementary Fig. 11d, Supplementary Fig. 11e, Supplementary Fig. 11g, Supplementary Fig. 11h, Supplementary Fig. 12a, Supplementary Fig. 12b). However, surprisingly, KT-NE (i.v) demonstrated a moderate systemic or localized anti-obesity outcome compared with KT-NE (s.c) and KT-NE (i.p) treatments, possibly due to its relatively lower accumulation in adipose tissues (Fig.  8c, e). Furthermore, all KT-NE-treated groups had dramatically smaller adipocytes compare to untreated mice with obesity (Fig. 9j), particularly in SAT of KT-NE (s.c) and adipocytes in VFD of KT-NE (i.p) (Fig. 9k, l).

Several evidences have suggested a mechanistic link between obesity and NAFLD, with visceral fat being considered a key contributor to steatohepatitis^49^. Detailed tissue analysis revealed that KT-NE-treated groups (particularly KT-NE (s.c) and KT-NE (i.p) strategies) had significantly reduced liver mass (Fig. 9m) and ameliorated hepatic steatosis (Fig. 9n, Supplementary Fig. 10e, f), exhibiting substantially less lipid accumulation and deposition in livers according to various histological examinations (Supplementary Fig. 13). Furthermore, KT-NE-treated mice, particularly these who accepted s.c. and/or i.p. injection, appeared to have lower ROS level and XBP1 expression in liver (Supplementary Fig. 10c, Supplementary Fig. 10f, Supplementary Fig. 10i, Supplementary Fig. 10l). Additionally, KT-NE-treated female mice with obesity demonstrated lower content of triglycerides, AST and ALT in serum (Fig. 9o–q), suggesting that KT-NE treatment effectively mitigated the elevation of NAFLD biomarkers associated with obesity.

In summary, these findings suggest that KT-NE administration can delay or inhibit obesity progression and effectively impede the development of NAFLD, even in the presence of a persistent HFD. Moreover, the choice of administration routes significantly affected the localized and systemic anti-adiposity effects of KT-NE, with s.c. multi-point administration favoring SAT reduction and i.p. injection favoring systemic anti-adiposity outcomes.

## Discussion

Efficient strategies for combating localized or systemic obesity must adhere to specific principles: (1) ensuring safe biocompatibility and negligible side effects; (2) effectively restricting adipose tissue expansion in diverse populations; (3) preventing obesity-associated comorbidities such as NAFLD. However, currently available FDA-approved systemic anti-obesity medications mainly work through manipulating CNS pathway or through GI tract to disturb fat absorption^9^, which may provoke nervous system-associated stimulant- or depressant-like syndrome and increase kidney metabolic burden[9, 16]. In addition, FDA-approved localized anti-obesity drugs may induce non-negligible tissue necrosis and fibrosis adjacent to injection sites due to their surfactant-like indiscriminate cell membranes lysis^18,19^. Hence, there is an urgent need to explore novel strategies that offer high pharmacological safety and impactful anti-fat efficacy, especially considering the multifaceted physiological or pharmacological “lipogenic” stimuli worldwide.

**Fig. 10:**
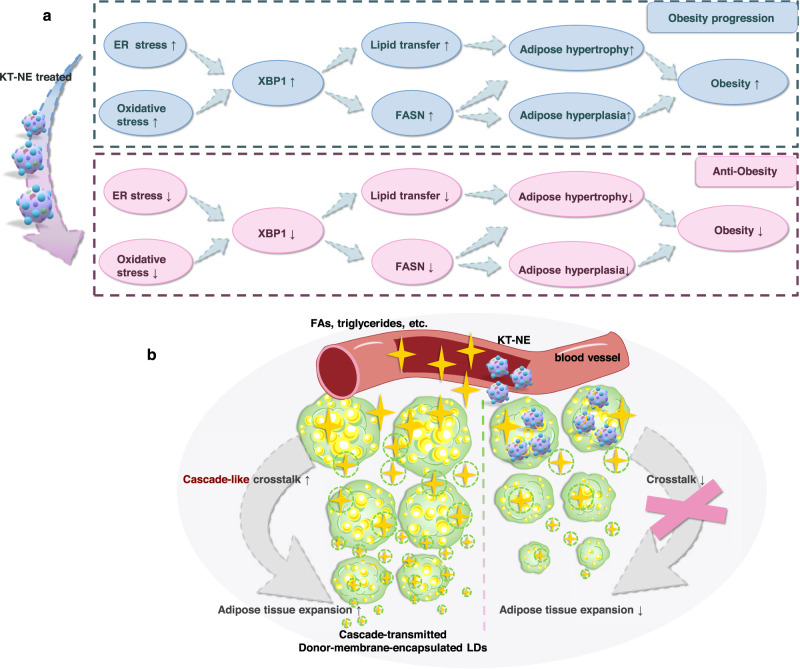
Schematic outline illustrating the mechanisms underlying anti-obesity efficacy of KT-NE. **a** KT-NE directly impeded the adipose progression, reliant on FASN-mediated adipogenesis, by downregulating XBP1s in (pre)adipocytes with KT-NE internalization. **b** KT-NE indirectly restricted adipose development, reliant on the interplay or transfer of lipid droplets with active adipocytes, by inhibiting the cascaded transmission of adipogenic signals in distal adipocytes and preadipocytes without KT-NE internalization.

Given that ROS and IRE1α-XBP1 pathway regulate physiological process of adipogenesis in vivo and in vitro^21,22,26,27,37^, we developed an oxidative stress- and ER stress- remission nanosystem called KT-NE to block the expansion of adipose tissues in individuals with obesity targeting adipose hyperplasia and hypertrophy (Fig. 10).

It is worth mentioning that lipid droplets grow dramatically after their initial formation, accelerating adipose hypertrophy in fatty tissues. Matured adipocytes typically feature several large lipid droplets within a relatively confined cellular space (Fig. 2b), where the volume of these droplets determines cell size. Although the mechanisms behind lipid droplet formation and enlargement remain largely unexplored, emerging evidence suggests two primary mechanisms: (1) direct enlargement or growth by incorporating newly synthesized fatty acids (or triglycerides) into expanding lipid droplets; (2) fusion, absorption or coalescence of nearby lipid droplets (from intercellular or intracellular lipid droplets)^50,51^. The former is closely associated with the physiological function and expression levels of FASN and other triglyceride synthesis isoenzymes. The latter may be positively correlated with lipid droplet transfer (or intercommunication) among adipocytes, dependent on adipocyte membrane fluiditys^39,52,53^. In addition, the enlargement in fat depots occurs in a Domino-like cascaded adipose as the adipose hyperplasia or hypertrophy of (pre)adipocytes are linked to the transmission and diffusion of adipogenic signal, including lipid droplets and other adipocytokines from the adipocytes approaching blood vessels^38^ (Fig. 10b).

Therefore, for adipocytes and preadipocytes internalizing KT-NE, our nanosystem could directly hinder adipose hyperplasia or hypertrophy, which largely depends on FASN (and other triglyceride synthesis isoenzymes)-mediated adipogenesis, achieved by downregulating XBP1s (Fig.10a). Additionally, for distal adipocytes and preadipocytes without KT-NE internalization, our nanosystem could indirectly impede adipose development, primarily reliant on intercommunication or lipid droplet transfer with active adipocytes, thus restricting the cascaded transmission of adipogenic signals both in vivo and in vitro (Fig. 10b). In short, KT-NE demonstrated significant anti-obesity efficacy by hindering triglyceride biosynthesis and impeding the cascade-like progression of adipose tissue.

Furthermore, KT-NE could effectively control or decrease body weight (fat) and maintained organismal fitness and homeostasis, potentially even in individuals with obesity under a high-calorie diet. In clinical practice, interventions for weight or fat loss necessitate a multimodal comprehensive approach that includes lifestyle changes (e.g., adopting a healthier diet) along with drug treatments. However, changing habitual lifestyle behaviors can be challenging and demanding. Therefore, our approach might offer an alternative means to control body weight and prevent obesity-related complications, especially for patients with obesity who struggle to maintain lifestyle modifications. In summary, our stress-relief nanosystem KT-NE may serve as a valuable reference for future clinical development of obesity pharmacotherapy.

Furthermore, systemic administration routes (i.v. and i.p.) and localized administration (s.c.) lead to different localized and systemic anti-adiposity effects. Surprisingly, the vast majority of KT-NE administered via i.v. injection accumulated in liver and lung rather than in widely distributed fatty tissues and KT-NE (i.v.) showed less anti-obesity effect compared to the other two methods. However, oral administration is the most common delivery route in current pharmacotherapies for obesity^9^. Drugs distributed into target tissues are generally fewer in oral administration due to the liver’s first-pass effect, which might partially explain why existing anti-obesity pharmacotherapies exhibit disappointing clinical outcomes but have a low degree of hepatotoxicity. In this context, optimizing or altering administration routes for obesity therapy could be a crucial area for future exploration.

In conclusion, our study presents a strategy to alleviate the burden of obesity and reduce the risk of adiposity-related complications (e.g., NAFLD) by an ER stress- and oxidative stress- relieving nano-platform KT-NE to scavenge excessive ROS and restrain XBP1 hyperactivation in adipose tissues. Moreover, KT-NE could inhibit cascaded adipose hyperplasia and hypertrophy by restricting adipocyte interaction, mainly lipid droplets transfer, and blocking triglyceride biosynthesis in vivo and in vitro. Additionally, the choice of administration routes significantly influenced the biodistribution and localized and/or systemic anti-obesity effects of KT-NE. Overall, the therapeutic strategy proposed in our study might be a promising approach for the development of anti-obesity treatments.

## Methods

All experiments were carried out according to the requirements of the Zhejiang University Animal Study Committee (approval no.23044).

### Materials and reagents

α-Tocopherol, dexamethasone (DEX), 3-isobutyl-1-methylxanthine (IBMX), and insulin were obtained from Sigma-Aldrich (Merk, Darmstadt, Germany). Olive oil (CAS:8001-25-0) and glycerol trioleate (CAS:122-32-7) were purchased from Aladdin (Shanghai, China). Phosphatidylcholine (PC) PL100M was obtained from Kewpie Co. (Shibuya, Japan). Medium-chain triglyceride (MCT) was gained from Lipoid (Merk, Darmstadt, Germany). KIRA6 (CAS: 1589527-65-0) was acquired from MedChemExpress (Monmouth Junction, NJ, USA). XBP1s rabbit polyclonal antibody was sourced from proteintech (Cambridge, UK, 24868-1-AP). Fatty Acid Synthase rabbit polyclonal antibody from proteintech (Chicago, USA, Cat No: 10624-2-AP) and rabbit anti-β-actin antibody from proteintech (Chicago, USA, Cat No: 81115-1-RR). 3,3′-dioctadecyloxacarbocyanine perchlorate (DIO, CAS: 34215-57-1), 1,1′‐dioctadecyl‐3,3,3′,3′‐tetramethylindodicarbocyanine perchlorate (DID, CAS: 127274-91-3), and 1,1-dioctadecyl-3,3,3,3-tetramethylindotricarbocyaine iodide (DIR, CAS: 100068-60-8) were purchased from Meilun Biotech Co. Ltd. (Dalian, China). Cell Counting Kit-8 (CCK-8), ROS detection kit (2’,7’-Dichlorodihydrofluorescein diacetate, DCFH-DA, CAS: 4091-99-0), and lipid droplet detection kit (4,4-Difluoro-1,3,5,7,8-Pentamethyl-4-Bora-3a,4a-Diaza-s-Indacene, BODIPY 493/503, CAS: 121207-31-6) were purchased from GLPBIO (USA). Mouse Triglyceride (TC) enzyme-linked immunosorbent assay (ELISA) kits were obtained from Jiangsu Meimian Industrial Co., Ltd (Jiang Su, China). 2’-(4-ethoxyphenyl)-6-(4-methylpiperazin-1-yl)-1H,3’H-2,5’-bibenzo[d]imidazole trihydrochloride (Hoechst 33342, CAS: 23491-52-3), RIPA lysis buffer, protein loading buffer (5X), BCA protein assay kit, polyvinylidene fluoride (PVDF) membranes, HRP-anti-rabbit IgG (H + L) (Beyotime, A0208), and protease inhibitor cocktail were obtained from Beyotime Institute of Biotechnology (Jiangsu, China). FITC-conjugated HRP-anti-rabbit IgG (H + L) was obtained from Hangzhou Dawen Biology Co., Ltd (Hangzhou, China). Dulbecco’s Modified Eagle Medium (DMEM), penicillin/streptomycin (100 U·mL^−1^), fetal bovine serum (FBS), and 0.25% trypsin with 0.02% ethylenediaminetetraacetic acid (EDTA) were purchased from Gibco (Thermo Fisher Scientific, USA). The deionized water used was prepared utilizing a Milli‐Q system (Millipore, Boston), and all reagents were of analytical grade.

### Cell lines and animals

The preadipocyte cell line 3T3-L1 was purchased from American Type Culture Collection (ATCC®, CL-173™). Primary umbilical vein endothelial cells (HUVEC) (ATCC®, PCS-100–010™), human embryonic kidney cells HEK-293 (ATCC®, CRL-1573™), immortalized keratinocytes HACAT, normal liver cells L-O2 (BeNa Culture Collection, Beijing, China, BNCC360120), and mouse C2C12 myoblasts cells (BeNa Culture Collection, Beijing, China, BNCC338573) were maintained at 37 °C in a humidified atmosphere with 5% CO_2_ (Heraeus, Germany). The cells were cultured in DMEM medium (Gibco, Thermo Fisher Scientific, USA), supplemented with 10% FBS, and 100 U/mL penicillin/streptomycin for fewer than 10 passages to preserve optimal cell viability and inherent characteristics.

The adipocytes used in the in vitro experiments were derived from 3T3-L1preadipocytes following an established protocol^34^. Briefly, 3T3-L1 preadipocytes were cultured in an appropriate medium until they reached contact inhibition for 2 days. Adipogenic differentiation was initiated in confluent cells by sequentially replacing the DMEM medium with differentiation media, including Inducer I (comprising 0.5 mM IBMX, 10 μg/mL insulin, and 0.5 μM DEX) and Inducer II (comprising 10 μg/mL insulin), in DMEM supplemented with 10% FBS and 100 U/mL penicillin-streptomycin. After 2 days of sequential exposure to differentiation media containing Inducer I and Inducer II, the cells were transitioned to maintenance media (DMEM complete medium containing 10% FBS and 100 U/mL penicillin-streptomycin) for an additional 6–8 days until mature adipocytes formed. The culture medium was replenished with fresh medium every other day. Notably, the 3T3-L1 preadipocytes stimulated by differentiation media containing Inducer I were called Inducer I treated preadipocytes here, and those stimulated successively by Inducer I and Inducer II were termed Inducer II treated preadipocytes (Fig. 2a). All cell lines underwent routine testing for mycoplasma contamination and were confirmed to be negative.

Female C57BL/6 mice aged 8–10 weeks (obtained from Slaccas Experimental Animal Co., Ltd., Shanghai, China) were conventionally bred and housed in a pathogen-free environment. These mice underwent a 4-week high-fat diet (FBSH Biotechnology Co., Ltd., D12492, containing 60% fat, 4.75 Kcal/g) to induce obesity. The mice were maintained under a standard laboratory conditions at 22–25°C, with humidity maintained between 30% and 70%, and a 12 h light/dark cycle (lights on from 9 am to 9 pm, and lights off from 9 pm to 9 am). Mice were randomly assigned to different experimental groups. All in vivo experimental procedures were performed following protocols approved by the Institutional Animal Care and Use Committee of Zhejiang University.

### Construction, characterization and cytotoxicity of nanoemulsions

The KIRA6-loaded α-Tocopherol nanoemulsion (KT-NE) was formulated using the consecutive probe-type sonication method. In essence, PC, MCT, and α-Tocopherol were dissolved in ethanol in a 6:5:1 weight ratio, constituting the oil phase. The fat-soluble compound KIRA6 was incorporated into the oil phase prior to the nanoemulsion formation. Sequentially, Milli‐Q water, acting as the aqueous phase, was slowly dropped into the oil phase with vigorous stirring via vortex, resulting in the production of a primary oil-in-water nanoscale emulsion. The KT-NE formulation was then manufactured by subjecting the primary emulsion to probe sonication (35% power, work 2 s, pause 3 s, 3 min, 1–2 rounds) on an ice bath. Notably, the concentration of KT-NE utilized in the in vitro experiments was 1 µM, equivalent to the concentration of KIRA6. Additionally, three other nanoemulsions were yielded using the same method: Blank-nanoemulsion (Blank-NE, where KIRA6 and α-Tocopherol were replaced with MCT), α-Tocopherol nanoemulsion (T-NE, where KIRA6 was substituted with MCT), and KIRA6-nanoemulsion (K-NE, where α-Tocopherol was replaced by MCT).

To assess the encapsulation efficiency and loading capacity, an ultrafiltration-centrifugation and high-pressure liquid chromatography (HPLC)-based methodology was employed. Initially, ultrafiltration-centrifugation (15000 *g*, 10 min) was employed to separate the nanoemulsions into two fractions: the free drug part and the nanoemulsion part. Subsequently, both parts of the sample (free drug and nanoemulsion) were freeze-dried to remove water, and the weight of the nanoemulsion part was measured. Ethanol was then added to re-dissolve the lyophilized free drug and nanoemulsion parts, forming a solution containing KIRA6 and other lipid components. The content of the fat-soluble KIRA6 in the ethanol-based solution was determined using HPLC (HPLC 1200 series, Agilent Technologies). Specifically, the HPLC parameters were as follows: C18 Column (4.6 mm * 250 mm), mobile phase composition of 45% water (A) and 55% acetonitrile (B), a flow rate of 1 mL/min, and a detection wavelength of 330 nm. The encapsulation efficiency of KIRA6 in nanoemulsions was calculated using the formula ((Weight of KIRA6 in NEs)/(Weight of KIRA6 in NEs + weight of KIRA6 in medium) × 100%). Furthermore, the KIRA6 loading capacity in nanoemulsions was calculated using the formula ((Weight of KIRA6 in NEs)/(Weight of NEs) × 100%). For KT-NE, the encapsulation efficiency and loading capacity of KIRA6 were calculated to be 98.97% and 0.61%, respectively. Similarly, for K-NE, the values were 99.52% and 0.64%, respectively. These results indicated that the formulation of nanoemulsions, with or without α-tocopherol, did not significantly impact the drug loading capacity and encapsulation efficiency of the nanoemulsions.

The morphology of the nanoemulsions was observed by Transmission Electron Microscopy (JEOL JEM‐1230 microscopes, Japan), while their hydrodynamic sizes (Z-average) were assessed through Dynamic Light Scattering (Malvern Zeta sizer Nano‐ZS instrument, UK). To evaluate stability, nanoemulsions were stored appropriately at 4 °C, and any changes in particle size were monitored over 1 month. Additionally, the cytotoxicity of these preparations to various cell lines, including the (pre)adipocyte subgroups (3T3-L1 preadipocytes, Inducer I treated preadipocytes, Inducer II treated preadipocytes and adipocytes), as well as normal somatic cell lines (HUVEC, HEK-293, L-O2, HACAT and C2C12), was determined using the CCK8 assay following the manufacturer’s instructions over a 24-h period.

### Cellular uptake to nanoscale emulsions

To evaluate the time-dependent internalization ability of four nanoemulsions (including KT-NE (containing 1 μM KIRA6), K-NE, T-NE, and Blank-NE), various cell lines, including (pre)adipocytes and normal somatic cells, were preseeded in a 24-well dish with a confluence of 60–70%, using 1 mL complete medium per well. The following cell lines were utilized: 3T3-L1 preadipocyte, Inducer I treated preadipocyte, Inducer II treated preadipocyte, adipocyte, HUVEC, HEK-293, L-O2, HACAT, and C2C12. Fluorescent dye DID-labeled nanoemulsions (25 μg/mL DID) were administered to the cells for varying periods within 24 h. Simultaneously, Hoechst 33342 (10 μg/mL, 20 min, 37 °C, in the dark) was used to stain the nuclei. Fluorescence images were captured at 2 h, 6 h, 12 h, 16 h, and 24 h, employing an inverted high-resolution fluorescence microscopy (ECLIPSE Ti, C-HGFI, Nikon) under constant laser intensity. Different fluorescent images within the same field of vision were merged using the EZ-MET software.

### Visualization of KT-NE release characteristics in adipocytes

To visualize the release behavior of KT-NE in adipocytes within a 24-h timeframe, fat-soluble dyes DID (red, 25 μg/mL) and DIO (green, 25 μg/mL) were utilized. DID marked the hydrophobic core of the nanoemulsion, while DIO served as a model drug to mimic KIRA6 release. Fluorescence images were captured at three distinct time points (2 h, 10 h, and 24 h) using high-resolution fluorescence microscopy (ECLIPSE Ti, C-HGFI, Nikon) under constant laser intensity. The lack of full co-localization between red fluorescence and green fluorescence within adipocytes indicated drug release from KT-NE.

### Assessment of intracellular lipid droplets and ROS levels

To assess intracellular lipid droplet content, we employed the fluorescent dye BODIPY 493/503. Indicated (pre)adipocytes were stained with BODIPY 493/503 at a concentration of 5 μM in PBS buffer solution for 20 min at 37 °C, away from the light. Nuclei were counterstained with 10 μg/mL Hoechst 33342, and any excess dye was subsequently removed through fresh PBS wash. For the detection of intracellular ROS levels in grouped (pre)adipocytes, we utilized the cell-permeable probe DCFH-DA at a concentration of 10 μM. This probe was applied for 20 min at 37 °C in the dark, and Hoechst 33342 was used for nucleic localization. Following staining procedures, high-resolution fluorescence microscopy (ECLIPSE Ti, C-HGFI, Nikon) was used to capture fluorescent images.

### Western blot, immunofluorescence and ELISA

For the extraction of indicated (pre)adipocytes and tissue lysates, ice-cold RIPA lysis buffer supplemented with phosphatase and protease inhibitor cocktail at an appropriate concentration was used. The lysates were then mixed with protein loading buffer (5X) and boiled at 95 °C for 10 min. Subsequently, the grouped samples with equal protein concentration were electrophoresed by SDS-PAGE, transferred onto methanol-pretreated PVDF membranes, and blocked with 5% bovine serum albumin (BSA) at 37 °C for 2 h. After being washed thrice, protein bands were incubated overnight at 4 °C with the corresponding primary antibodies. They were then incubated for 2 h at 37 °C with secondary antibodies conjugated with HRP. Ultimately, the protein bands were detected using an enhanced chemiluminescence system (Bio-Rad). The antibodies utilized included rabbit anti-XBP1 (1:1000), FASN (1:1000), β-actin (1:1000), and HRP-anti-rabbit IgG (H + L) (1:2000).

The indicated (pre)adipocytes were initially pre-seeded in a 48-well plate, fixed with 4% formaldehyde for 10 min, and then blocked with DMEM medium containing 5% FBS. Subsequently, the cells were incubated with specific primary antibodies for 2 h at 37 °C, followed by three washes with PBS to remove unconjugated antibodies. The cells were then incubated with FITC-conjugated HRP-anti-rabbit IgG (H + L) for 2 h at 37 °C in the dark. Afterward, the cells were stained with Hoechst 33342 to mark nucleus, and underwent a final wash to remove excess dyes. The resulting immunofluorescence images were captured using high-resolution fluorescence microscopy (ECLIPSE Ti, C-HGFI, Nikon). The antibodies used here included rabbit anti-XBP1 (1:100), FASN (1:100), and FITC-conjugated HRP-anti-rabbit IgG (H + L) (1:100).

Peripheral blood samples were meticulously collected from mice with obesity induced by a HFD. After overnight storage at 4 °C, the blood samples underwent centrifugation at 400 *g* for 10 min to obtain the upper serum. The concentration of glycerin trilaurate in these serum samples was then determined using ELISA kits, following the detailed instructions provided by the manufacturer.

### Lipid droplet transfer between the donor and the recipient

To elucidate the dynamics of lipid droplet transfer among adipocytes, encompassing both contact-dependent and non-contact-dependent mechanisms, we used a dual-labeling approach to mark the donor and the recipient adipocytes. In detail, the adipocytes pre-labeled with membrane-visualized dyes DID (red) served as the donor of lipid droplets, while the adipocytes pre-marked with fluorescent dyes DIO (green) acted as the recipient of lipid droplets.

Following treatment with the indicated nanoemulsions (T-NE, K-NE, and KT-NE) for 24 h, DID-labeled donors and DIO-labeled recipients were co-cultured for an additional 24 h at a 1:1 ratio. Subsequently, high-resolution fluorescent images were acquired (ECLIPSE Ti, C-HGFI, Nikon). The membrane-visualized fluorescent dyes selectively targeted biofilm components, such as the plasma membrane, extracellular vesicle membrane, and even the lipid droplet membrane. In addition, lipid droplets, when transported to the extracellular space, likely had a coating of the donor’s membrane, resulting in DID-labeled membrane encapsulation. Therefore, the extracellular red dots observed in the images represented membrane-encapsulated small lipid droplets secreted by the DID-labeled donor adipocytes. The holes in both donor and recipient adipocytes indicated the locations of intracellular big lipid droplets. It is essential to note that successful lipid droplet transfer between the donor and recipient was confirmed when the red dots precisely filled the holes of the DIO-labeled recipient. The experiments were repeated three times independently, with similar results.

### Lipids absorption, transmission, and diffusion in adipose tissue in vivo

To assess transmission and diffusion of lipids within adipose tissue, female C57BL/6 mice, aged 8–10 weeks, were induced into obesity through a 4-week-HFD. Following pre-treatment with multi-point s.c. injections of various nanoemulsions (T-NE, K-NE, or KT-NE) for two doses within 1 week, the mice received s.c. multi-point injections of 100 μL DIR-labeled mixed lipids solution (MCT: olive oil: glycerol trioleate = 1:1:1). 48 h after administration, the mice were euthanized for subsequent diffusion assessment of mixed fluorescent lipids (Biospace, Optima). Notably, the primary metric for assessing in vivo lipid droplet transfer among adipocytes was the quantification of the transmitted and diffused area of mixed fluorescent lipids in SAT.

### Biodistribution of KT-NE

To track the in vivo distribution of KT-NE, we employed the fluorescent dye DIR, which is excitable by near-infrared (NIR) light. When female 8–10 week-old C57BL/6 mice were fed on for 4 weeks, the HFD-induced mice with obesity were randomly assigned to three groups. Subsequently, each group received injections of DIR-labeled KT-NE through different administration routes (i.v., i.p. and s.c.). After 48 h, the mice were humanely euthanized, and the biodistribution of DIR-marked KT-NE was assessed in SAT, VFD, and major visceral organs (Biospace, Optima).

### In vivo therapeutic anti-obesity strategies

#### Multi-point subcutaneous administration of nanoemulsions in mice with obesity

To evaluate the anti-obesity therapeutic efficacy of KT-NE, female 8–10 week-old C57BL/6 mice were subjected to an ad libitum HFD for 4 weeks to induce obesity. Subsequently, the female mice with obesity were randomly divided into five groups (*n* = 12 biologically independent animals in each group) and underwent multi-point s.c. administration near bilateral inguinal SAT. Notably, the administrations included phosphate-buffered saline (PBS) and indicated formulations, namely Blank-NE, T-NE, K-NE, KT-NE, and a reference formulation (10% deoxycholic acid, FDA-approved for local fat ablation). These administrations were conducted twice per week.

The administered dose of KT-NE was 4 mg/kg (equivalent concentration of KIRA6). Importantly, the female mice with obesity continually received an ad libitum HFD throughout the treatment period. All preparation solutions underwent sterilization through a 0.22 μm sterile microporous filter to eliminate pathogenic microorganisms. Body weight and abdominal perimeter were monitored every other day until the termination of the experiment. Furthermore, daily food consumption (g/day) was manually measured, and a conversion rate of 4.75 kcal/g was employed to calculate the daily calorie intake (kcal/day). Body fat distribution in mice with obesity was assessed by using MILabs micro-CT imaging system on day 1 and day 21. On the terminal day, mice were humanely euthanized by overdosing with Pentobarbital sodium (250 mg/kg, i.p.), and the SAT and VFD were collected for weight measurements. The relative adipose tissue indexes (total adipose tissue mass/mice body weight) were analyzed. Serum biochemical indicators (triglycerides, AST, and ALT) concentrations were determined using an ELISA kit and Automatic biochemical analyzer LW C400. The ROS content in SAT, VFD, and liver was detected by Thermo Scientific Varioskan Flash 5250040, detecting the relative fluorescence intensity of DCF (Dichlorofluorescein), along with immunofluorescence staining. The XBP1s protein expression in SAT, VFD and liver was detected through western blot and immunofluorescence staining.

#### Evaluation of KT-NE efficacy and biosafety in mice with obesity via various administration routes

To comprehensively assess the anti-obesity effects and biosafety of KT-NE, female C57BL/6 mice aged 8–10 weeks, rendered obesity through a 4-week high-fat diet (HFD), were subjected to identical doses of sterile KT-NE (4 mg/kg). The administration routes included i.v., i.p., and s.c. injections, with a frequency of twice per week. Obesity-related parameters such as body weight, abdominal perimeter, body fat distribution, and daily calorie intake were continually monitored, as detailed in section “4.11.1”. At the end of the study, all mice were euthanized through i.p. injection of an overdose of Pentobarbital sodium (250 mg/kg), and various biochemical indicators were assessed. SAT and visceral fat were isolated and weighed, and adipocyte size in adipose tissues was investigated by using H&E staining. Additionally, Oil Red O staining was used to evaluate lipid droplet contents in the liver. The major viscera, including heart, liver, spleen, lung, and kidney, were collected and subjected to H&E staining to evaluate the biocompatibility of treatment strategies.

### Statistical analysis

Data were represented as mean ± standard error. Statistical analyses, including unpaired or paired student’s *t*-tests for comparisons between two or several groups, were conducted as appropriate. All presented data are representative. Image J was used for the viewing, processing, and analysis of fluorescent images. To ensure robust analysis, experiments were independently performed at least three times, consistently yielding the same results. All statistical analyses were performed using Prism-GraphPad version 8.4 Software (San Diego, CA), with a value of *P* < 0.05 considered to be statistically significant.

### Reporting summary

Further information on research design is available in the Nature Portfolio Reporting Summary linked to this article.

### Supplementary information

Supplementary Information

Reporting Summary

### Source data

Source Data

## Data Availability

All data supporting the findings of the paper are available in the Supplementary Information file and in the. Source data are provided in this paper.
