# Supplementary material for: A nanoemulsion targeting adipose hypertrophy and hyperplasia shows anti-obesity efficiency in female mice
Source: Nat Commun. 2024 Jan 2;15:72. doi: 10.1038/s41467-023-44416-3 (PMC10761889; doi:10.1038/s41467-023-44416-3)
Supplement: Supplementary file 1 — Supplementary Information [file 41467_2023_44416_MOESM1_ESM.pdf]

Supplementary Information

A Nanoemulsion Targeting Adipose Hypertrophy and Hyperplasia Shows Anti-Obesity Efficiency in Female Mice

Yichao Lu[1#], Zhenyu Luo[1#], Huanli Zhou[1], Yingying Shi[1], Ying Zhu[1], Xuemeng Guo[1], Jiaxin Huang[1], Junlei Zhang[1], Xu Liu[1], Sijie Wang[1], Xinyu Shan[1], Hang Yin[1], Yongzhong Du[1], Qingpo Li[1*], Jian You[1,2,3,4*], Lihua Luo[1*]

Authors' Affiliations:

[1]College of Pharmaceutical Sciences, Zhejiang University, 866 Yuhangtang Road, Hangzhou, Zhejiang 310058, P. R. China

[2]State Key Laboratory for Diagnosis and Treatment of Infectious Diseases, 79 Qingchun Road, Shangcheng District, Hangzhou, Zhejiang 310006, P. R. China

[3]The First Affiliated Hospital, College of Medicine, Zhejiang University, 79 QingChun Road, Hangzhou, Zhejiang 310000, P. R. China

[4]Jinhua Institute of Zhejiang University, 498 Yiwu Street, Jinhua, Zhejiang 321299, P. R. China

[#] **These authors contributed equally:** Yichao Lu and Zhenyu Luo

*** Corresponding Author:**

Lihua Luo: Office: 086-0571-88981651; Email: luolihua@zju.edu.cn

Jian You: Office: 086-0571-88981651; Email: youjiandoc@zju.edu.cn

Qingpo Li: Office: 086-0571-88981651; Email: liqingpo@zju.edu.cn

Supplementary Fig. 1 Fabrication and characterization of nanoemulsions.

Supplementary Fig.2 KT-NE limits intercommunication among adipocytes in vitro.

Supplementary Fig.3 KT-NE limits lipids transmission and diffusion in adipose tissue in vivo.

Supplementary Fig.4 Establishment of high-fat-diet (HFD)-induced mice model with obesity.

Supplementary Fig.5 KT-NE (s.c.) boosts anti-obesity effect.

Supplementary Fig.6 KT-NE (s.c.) decreases ROS content and XBP1 expression in adipose tissues.

Supplementary Fig.7 KT-NE (s.c.) reduces XBP1s expression in subcutaneous and visceral adipose tissues.

Supplementary Fig. 8 KT-NE decreases ROS content and XBP1 expression in the liver of mice with obesity.

Supplementary Fig.9 Administration routes affect KT-NE biodistribution in mice with obesity.

Supplementary Fig.10 Delivery routes impact KT-NE anti-obesity effect in vivo.

Supplementary Fig.11 ROS content and XBP1 expression in adipose tissues and liver after treated by KT-NE.

Supplementary Fig.12 The XBP1 expression in adipose tissues after treatment with KT-NE.

Supplementary Fig.13 The XBP1 expression in liver after treated by KT-NE.

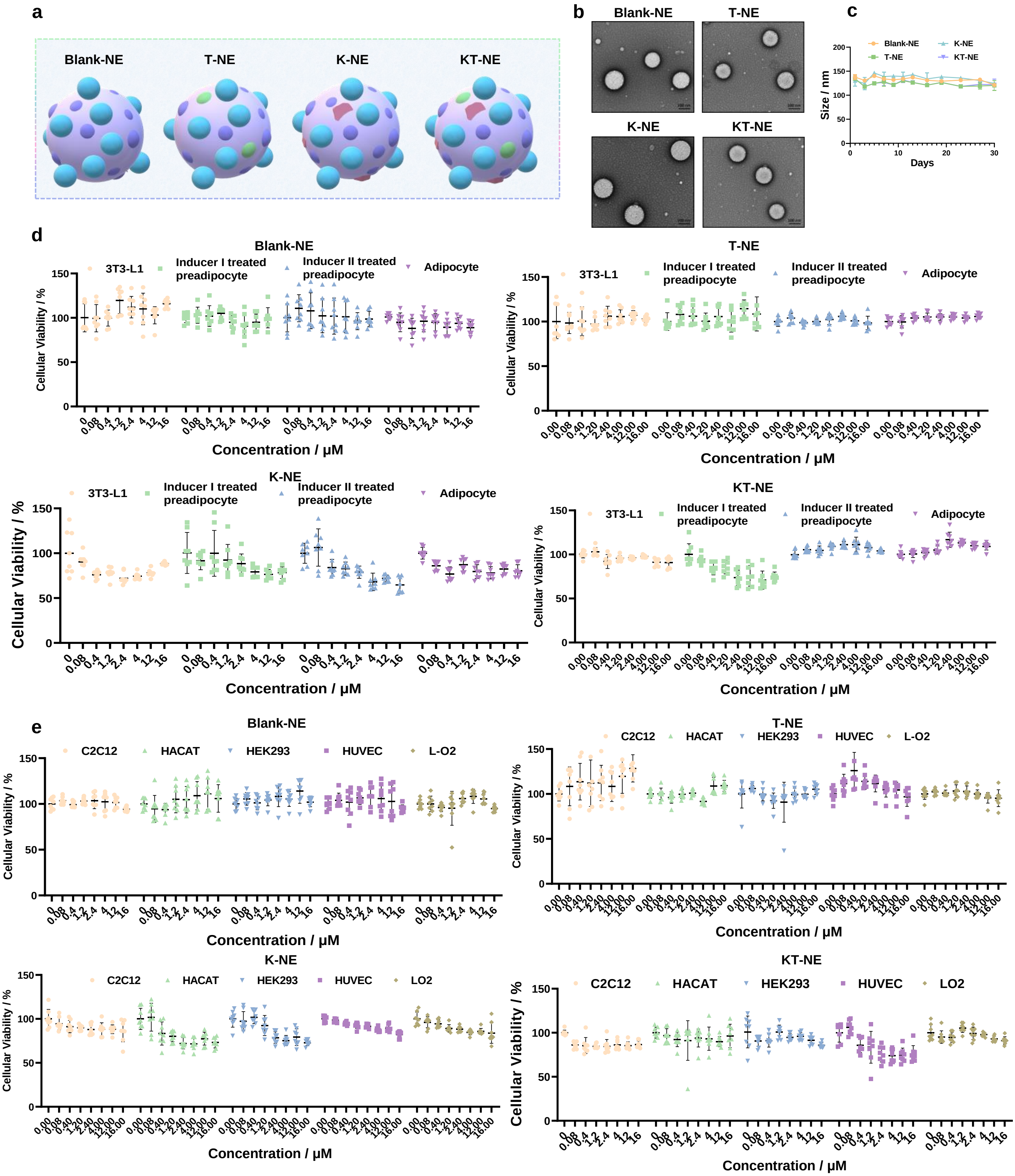

Supplementary Fig.1 Fabrication and characterization of nanoemulsions. (a) Abridged general view of compounds and a three-dimensional structure diagram of four nanoemulsions (NEs). **(b)** Morphologies of four NEs observed under transmission electron microscope (TEM). Scale bar = 100 nm. **(c)** Changes in the particle size of the indicated NEs within 1 month as stored under appropriate storage conditions at 4 °C, n =3. **(d)** Dose-dependent cell viability of indicated (pre)adipocytes (3T3-L1 preadipocytes, Inducer I treated preadipocytes, Inducer II treated preadipocytes, adipocytes) and **(e)** normal somatic cells (C2C12, HACAT, HEK-293, HUVEC, and L-O2) after) after incubation with indicated nanoscale emulsions at incremental drug concentration for 24 h, respectively, n = 8. All data was expressed as mean ± SD.

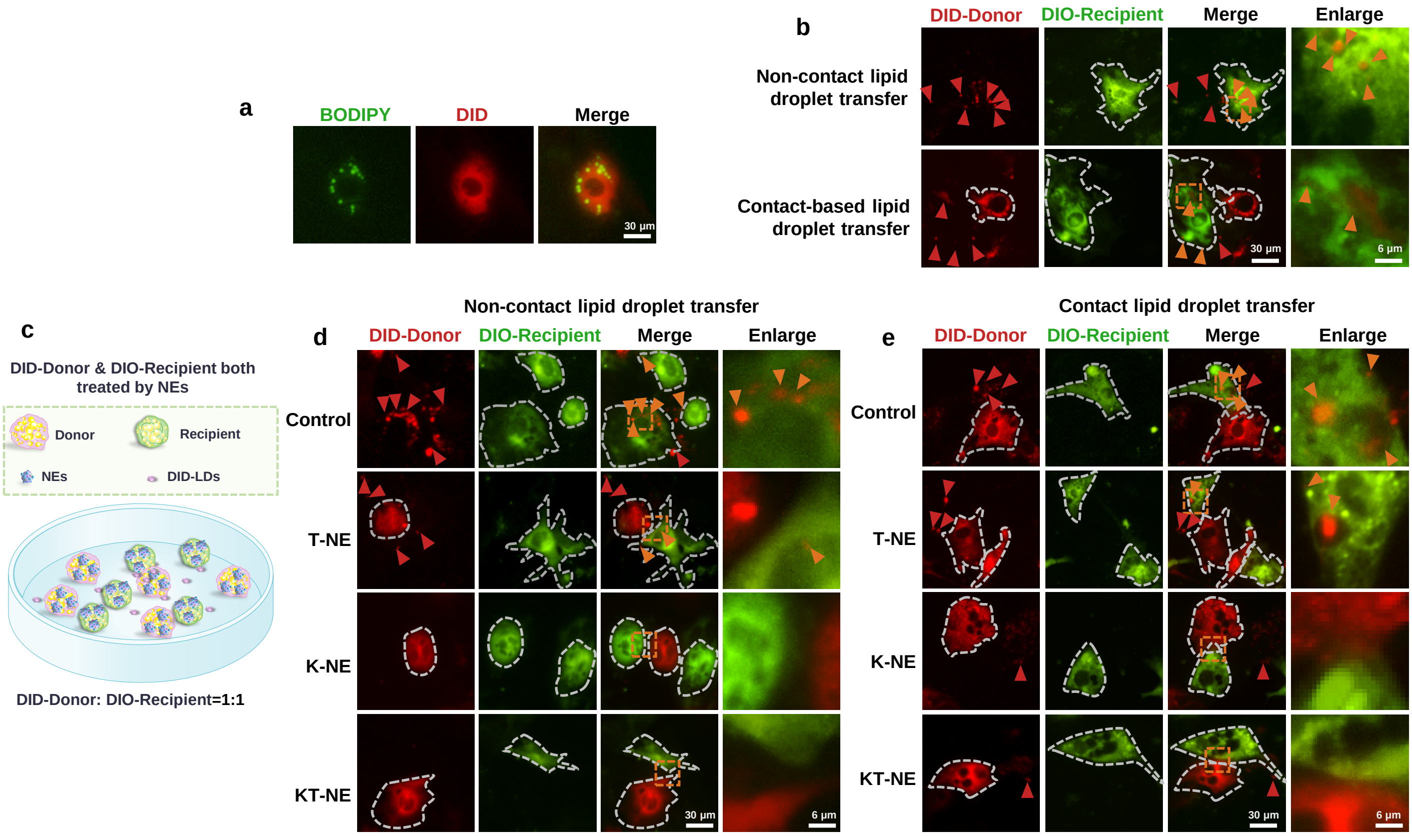

Supplementary Fig.2 KT-NE limits intercommunication among adipocytes in vitro. (a) Representative fluorescence images indicated that the holes in both donor and recipient adipocytes were where intracellular big lipid droplets were located. Scale bar=30 μm. **(b)** Representative fluorescence images demonstrated contact-based LDs transfer and non-contact-dependent LDs transfer between DID-labeled donor adipocytes and DIO-marked recipient adipocytes. The representative extracellular/intracellular red dots were DID-marked membrane-encapsulated small lipid droplets secreted by DID-marked donor adipocytes, highlighted with red triangles. The DID-marked membrane-encapsulated small lipid droplets internalized and accumulated into DIO-recipients (especially precisely into the big lipid droplets) were highlighted with orange triangles. Ttypical fields of vision were enlarged. Scale bar=30 μm, and scale bar=6 μm in enlarged images. **(c)** Schematic outline of co-culturing KT-NE-treated DID-labeled red donor adipocytes and KT-NE-treated DIO-labeled green recipient adipocytes. **(d)** Representative fluorescence images demonstrated non-contact LDs transfer and **(e)** contact-dependent LDs transfer between DID-labeled donor adipocytes and DIO-marked recipient adipocytes after treatment with KT-NE. Scale bar=30 μm, and scale bar=6 μm in enlarged images. Experiments were repeated three times independently, with similar results.

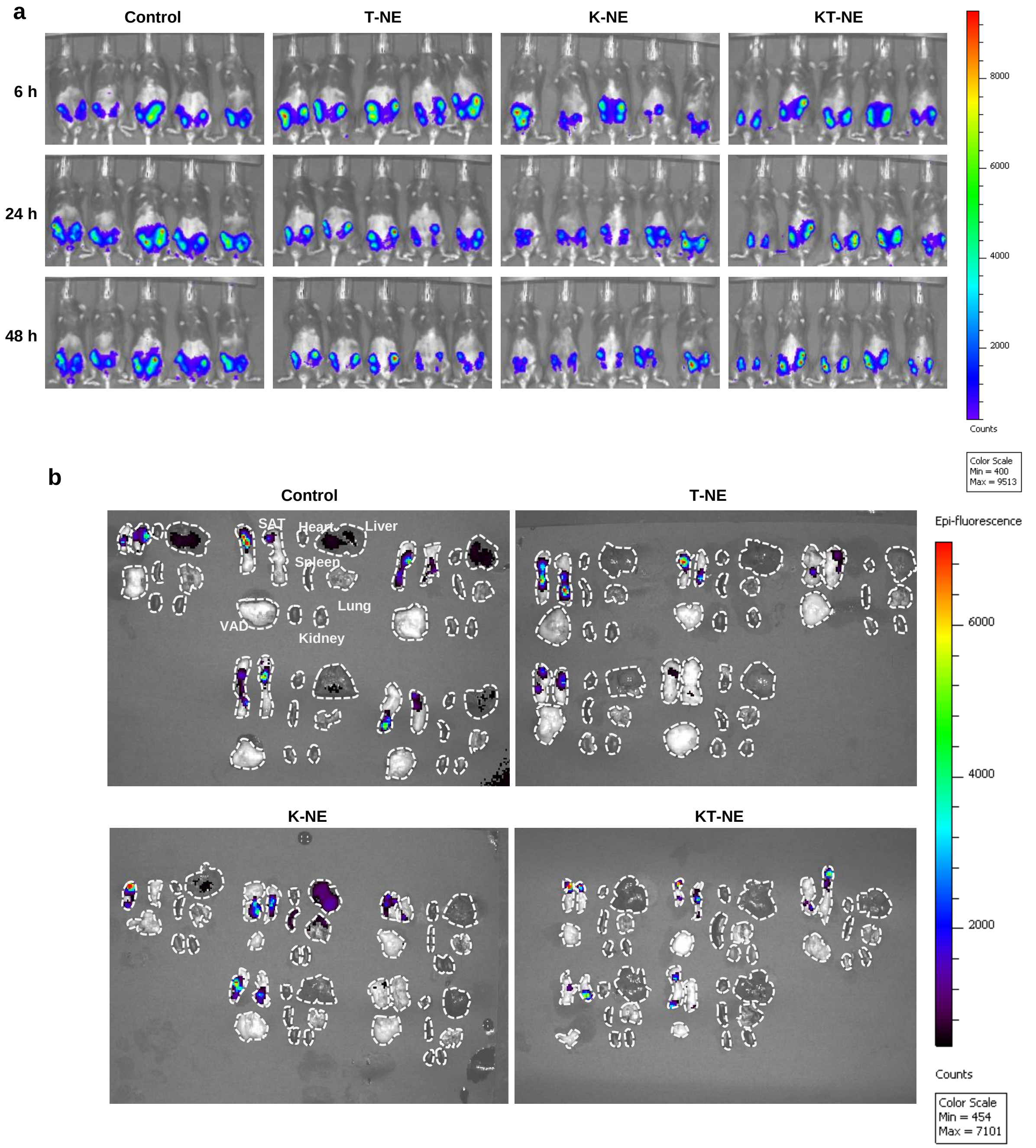

Supplementary Fig.3 KT-NE limits lipids transmission and diffusion in adipose tissue in vivo. (a) The biodistribution of DIR-labeled mixed lipids at 6 h, 24 h, and 48 h after indicated injection. **(b)** Biodistribution of DIR-labeled mixed lipids in subcutaneous adipose tissue (SAT), visceral fat depots (VFD), heart, liver, spleen, lung, and kidney of mice with obesity, after DIR-labeled mixed lipids injected (s.c.) into the hosts for 48 h.

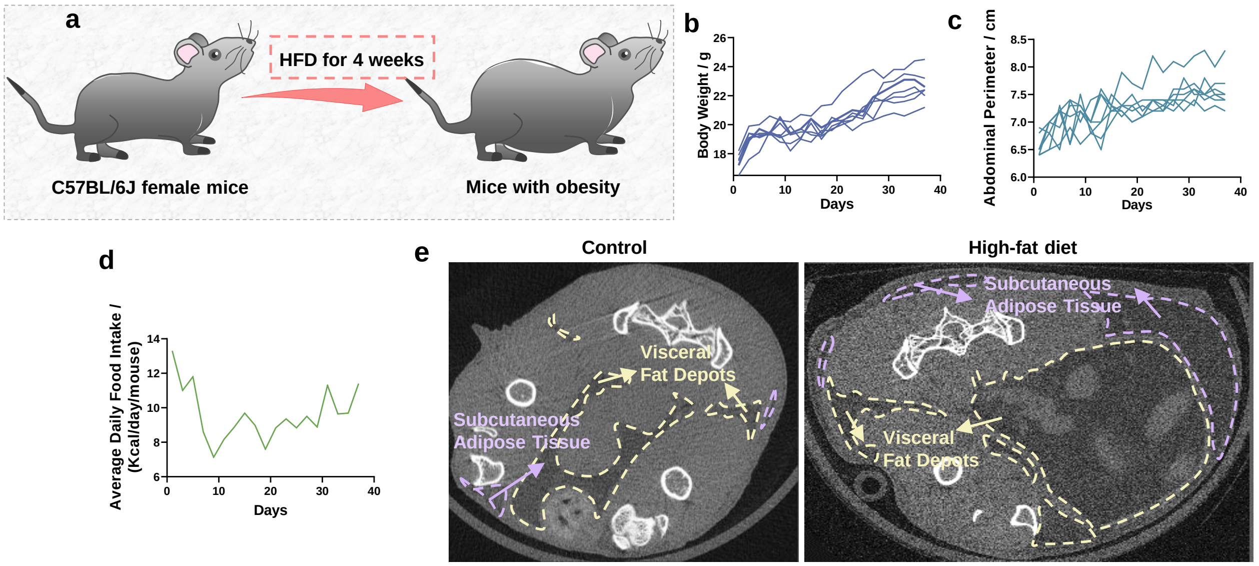

Supplementary Fig.4 Establishment of high-fat-diet (HFD)-induced mice model with obesity. (a) Schematic outline illustrated the procedure for establishing HFD-based mice model with obesity. **(b)** The body weight growth curve, **(c)** abdominal perimeter curve, and **(d)** average daily food intake curve of the mice during the construction of HFD-based mice model with obesity. **(e)** Micro-CT results to demonstrated the changes in body fat distribution in mice that were maintained on a HFD for 4 weeks. The darker areas represented represent regions of body fat distribution with lower density, where the subcutaneous adipose tissue was enclosed by the purple dotted line frame, and visceral fat depots were enclosed by the yellow dotted line frame.

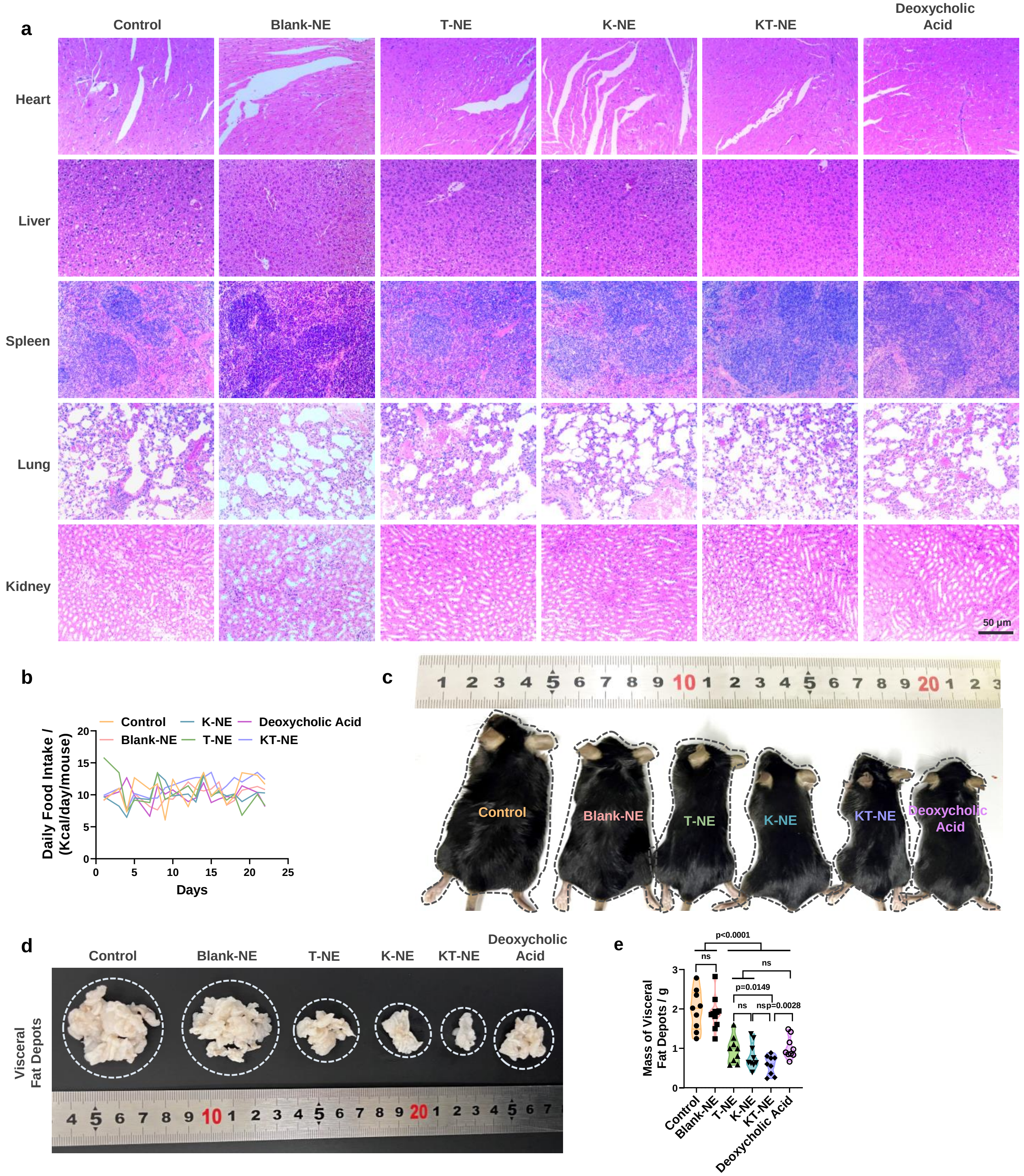

Supplementary Fig.5 KT-NE (s.c.) boosts anti-obesity effect. (a) Representative hematoxylin–eosin (H&E) staining images of heart, liver, spleen, lung, and kidney from indicated mice. Scale bar=50 μm. **(b)** Average daily food intake curve, and **(c)** representative digital image of indicated mice that received different preparations. **(d)** Representative digital image and **(e)** corresponding quantification diagrams of visceral fat depots from indicated groups. n=9. Statistical significance was evaluated by an unpaired two-tailed t-test. All data was expressed as mean ± SD, ns=no significance.

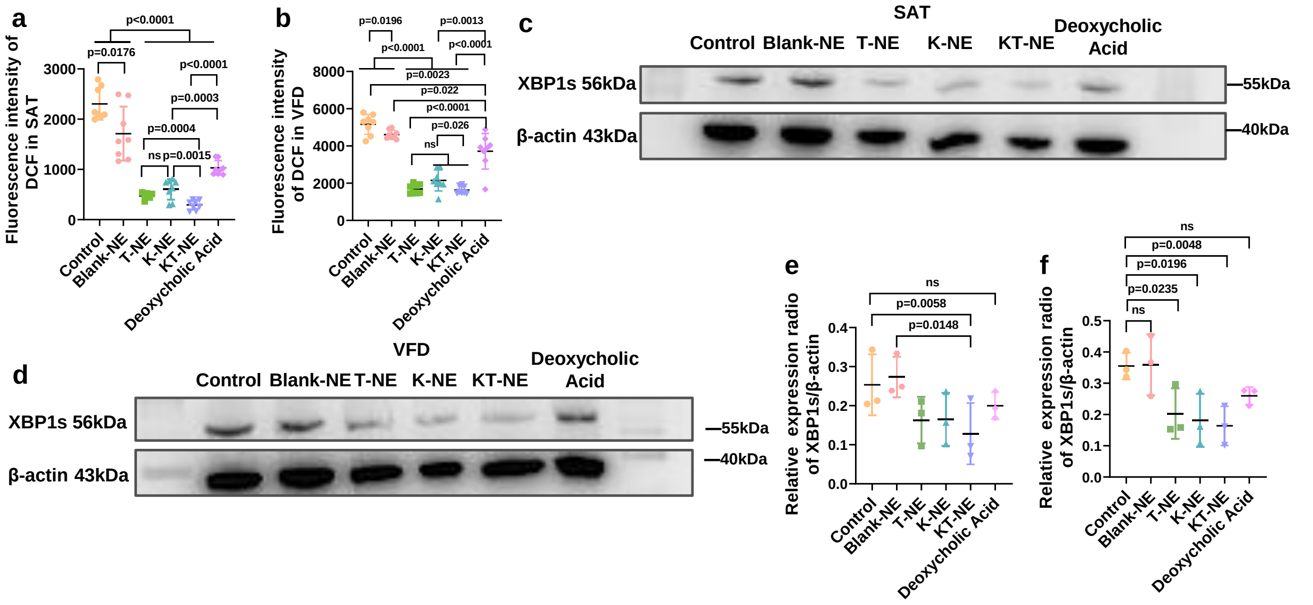

Supplementary Fig.6 KT-NE (s.c.) decreases ROS content and XBP1 expression in adipose tissues. (a) ROS content in adipose tissue (SAT) and **(b)** visceral fat depots (VFD) of indicated mice treated with different preparations. **(c-d)** Western blot results and **(e-f)** corresponding quantification (n=3) of XBP1s expression in SAT and VFD of the mice with obesity subjected to various treatment strategies. Experiments were repeated three times independently, with similar results. Statistical significance was evaluated by a paired two-tailed t-test All data was expressed as mean ± SD, ns=no significance.

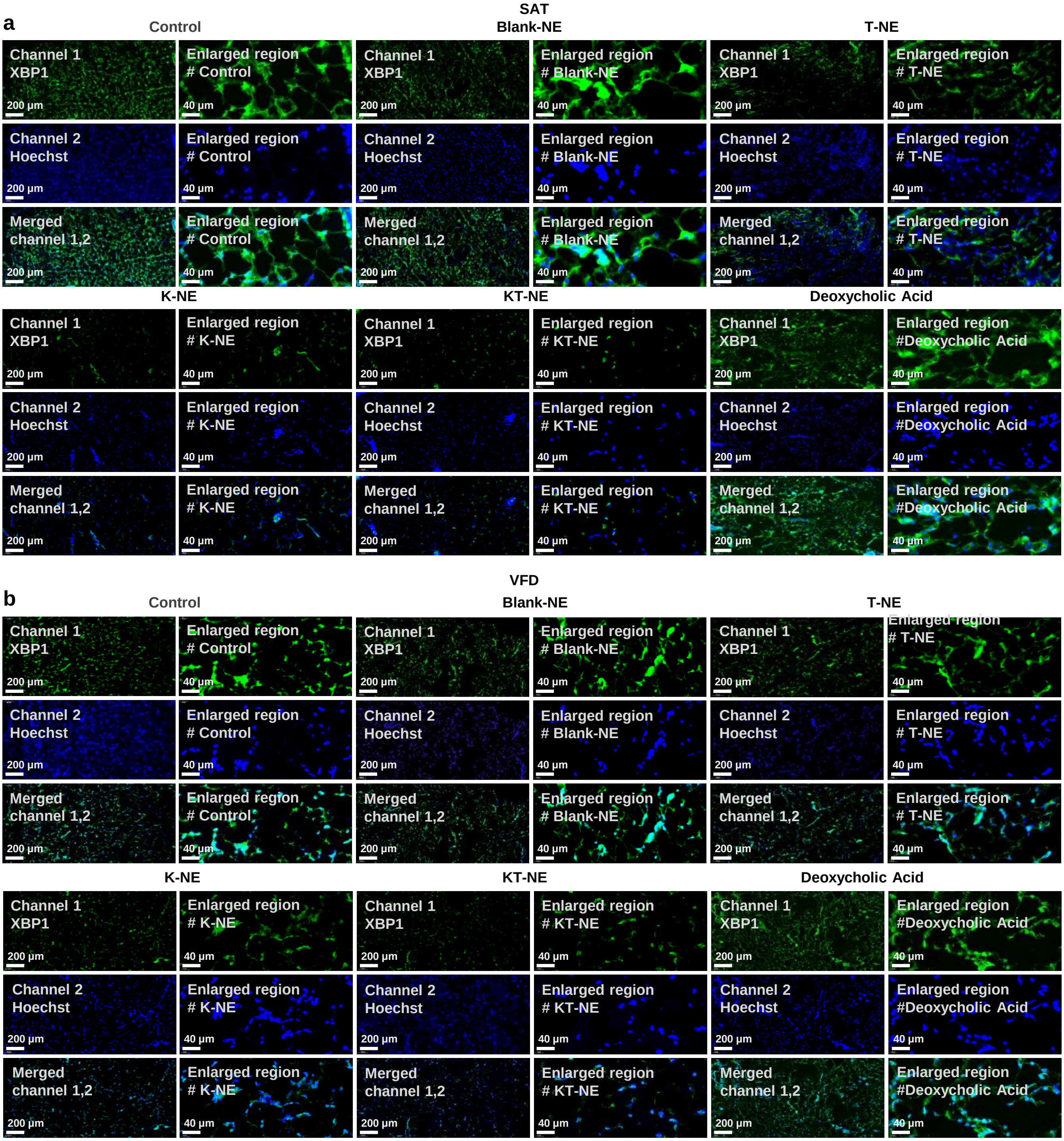

Supplementary Fig.7 KT-NE (s.c.) reduces XBP1s expression in subcutaneous and visceral adipose tissues. (a) Representative immunofluorescence images of XBP1s expression in subcutaneous adipose tissues (SAT) **(b)** and visceral fat depots (VFD) of the mice with obesity subjected to different treatment strategies. Scale bar=200 μm, and scale bar=40 μm in the enlarged images.

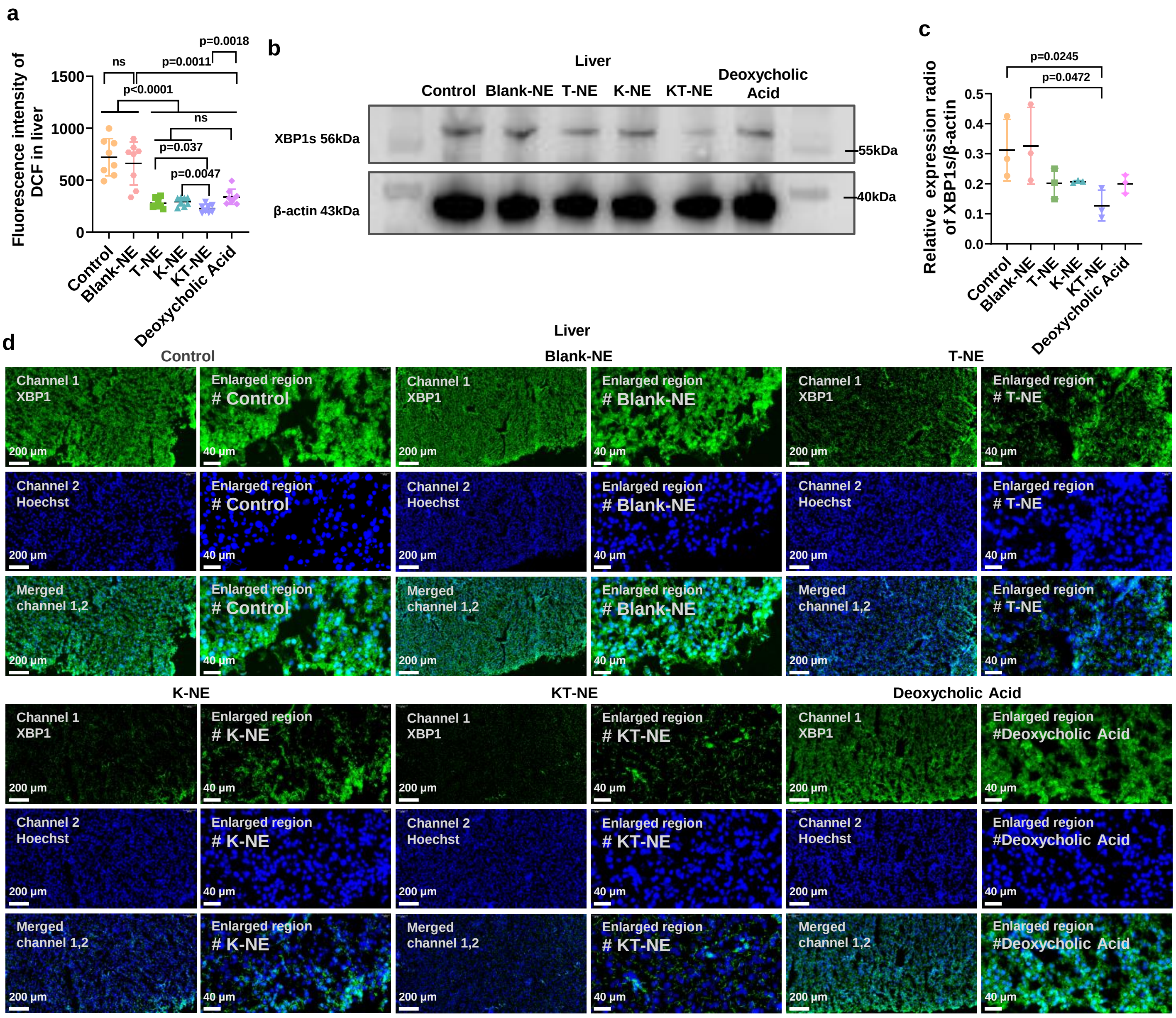

Supplementary Fig.8 KT-NE decreases ROS content and XBP1 expression in the liver of mice with obesity. (a) ROS content in the liver of indicated mice treated with different preparations. **(b)** Western blot results as well as **(c)** corresponding quantification (n=3) and **(d)** representative immunofluorescence images of XBP1s expression in the liver of the mice with obesity subjected to various treatments. Experiments were repeated three times independently, with similar results. Scale bar=200 μm, and scale bar=40 μm in the enlarged images. Statistical significance was evaluated by a paired two-tailed t-test. All data was expressed as mean ± SD, ns=no significance.

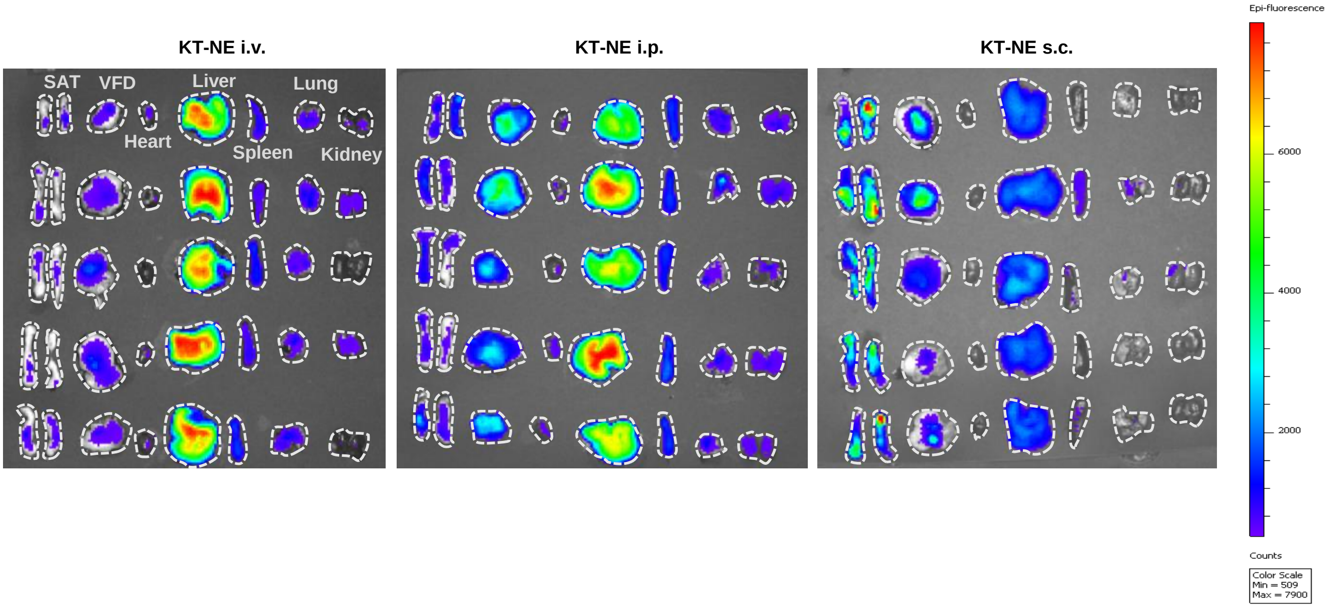

Supplementary Fig.9 Administration routes affect KT-NE biodistribution in mice with obesity. Fluorescence intensity of DIR-labeled KT-NE in various tissues, including subcutaneous adipose tissue (SAT), visceral fat depots (VFD), heart, liver, spleen, lung, and kidney of the mice with obesity. The assessments were taken 48 hours after the administration of KT-NE via different routes, including intravenous (i.v.), intraperitoneal (i.p.), and subcutaneous (s.c.) injections.

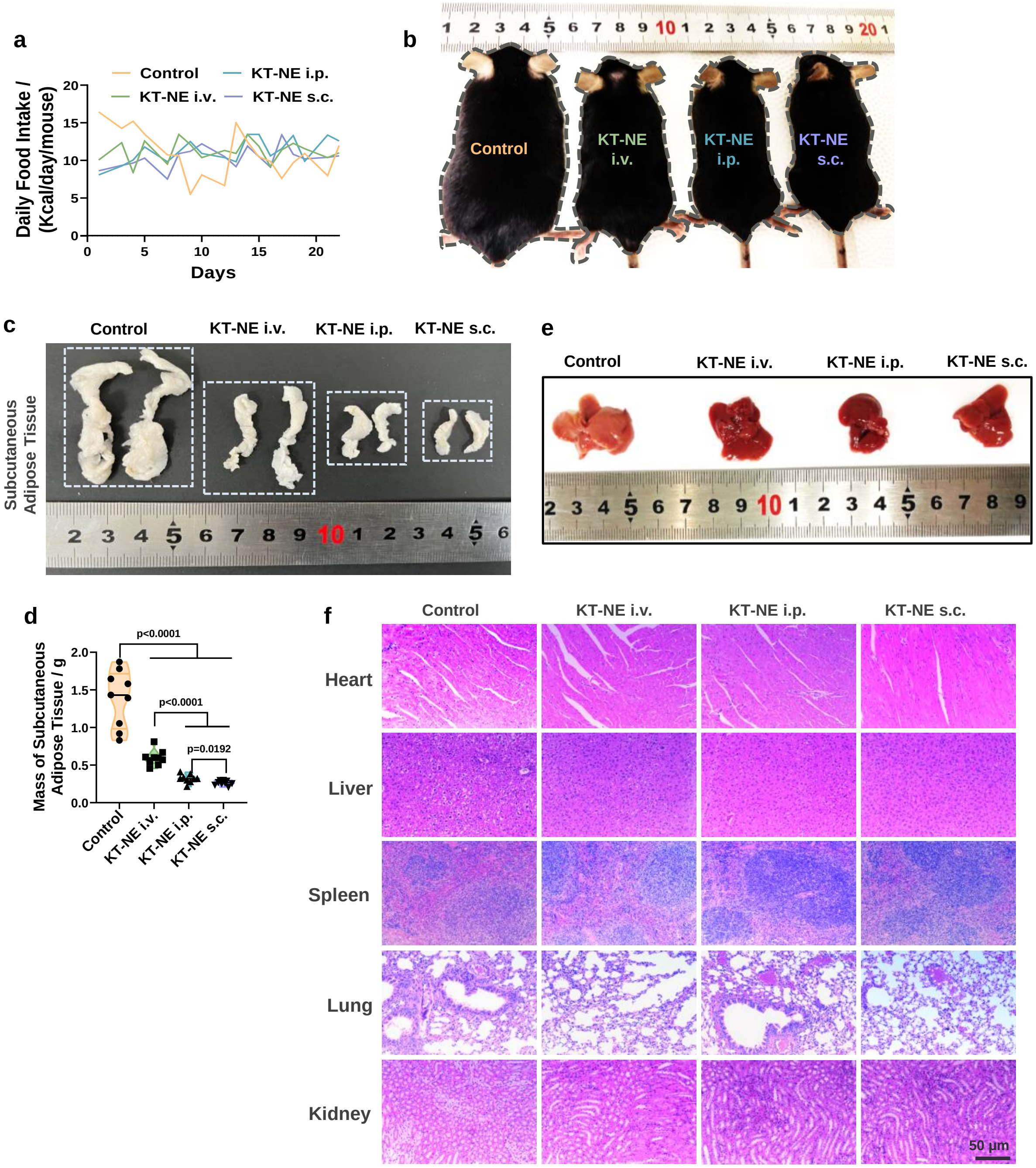

Supplementary Fig.10 Delivery routes impact KT-NE anti-obesity effect in vivo. (a) The average daily food intake curve of mice that received KT-NE treatment through intravenous (i.v.), intraperitoneal (i.p.), or subcutaneous (s.c.) injection. **(b)** A representative digital image of of the mice in the respective treatment groups. **(c)** Representative digital image and **(d)** corresponding quantification (n=9) of bilateral inguinal subcutaneous adipose tissue from the indicated groups. **(e)** A representative digital image of liver from indicated groups. **(f)** Representative hematoxylin–eosin (H&E) staining images of the heart, liver, spleen, lung, and kidney from the indicated mice. Scale bar=50 μm. Statistical significance was evaluated by an unpaired two-tailed t-test. All data was expressed as mean ± SD, ns=no significance.

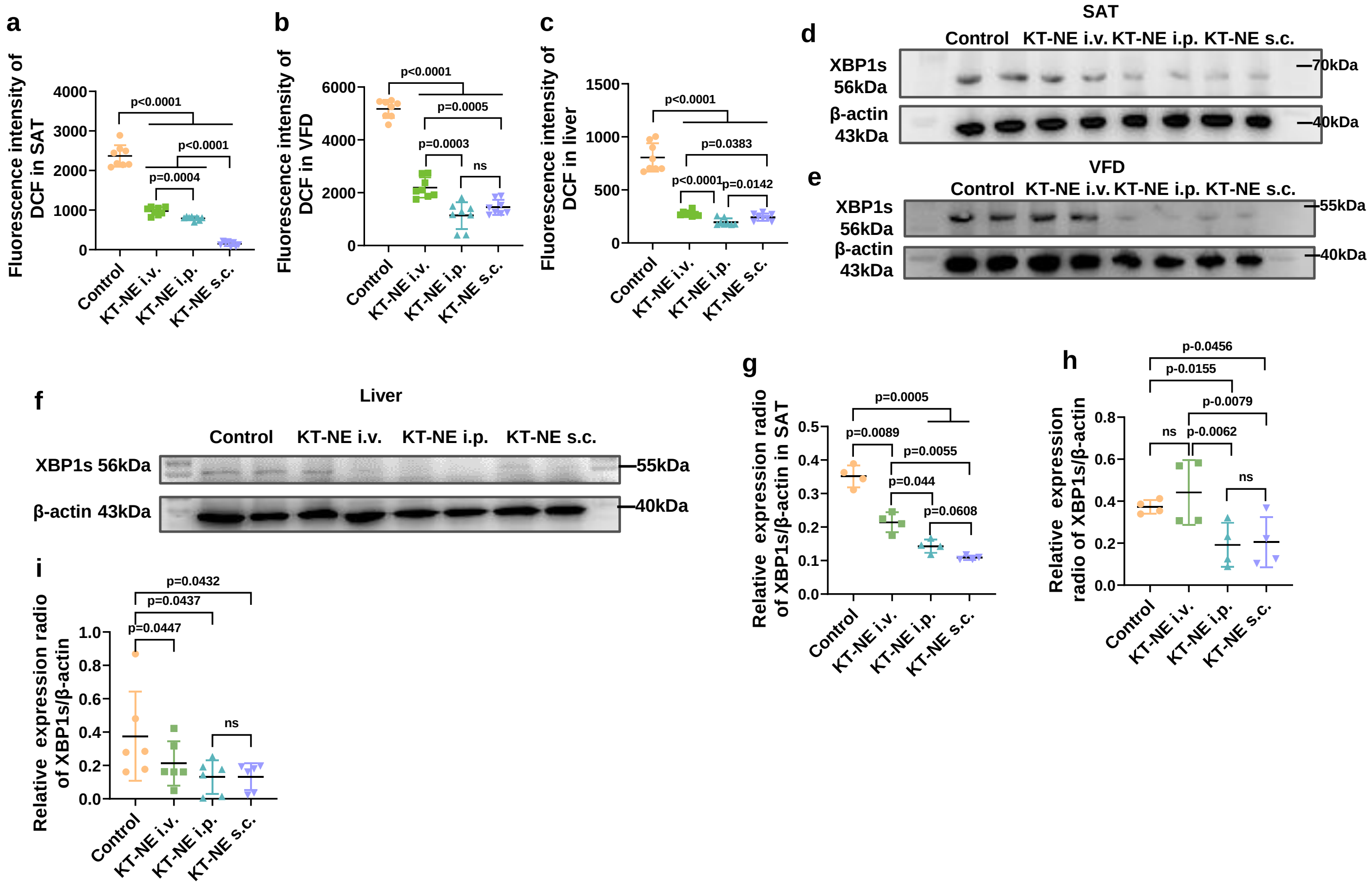

Supplementary Fig.11 ROS content and XBP1 expression in adipose tissues and liver after treated by KT-NE. (a) ROS levels in subcutaneous adipose tissue (SAT), **(b)** visceral fat depots (VFD), and **(c)** liver in the mice with obesity treated with KT-NE (i.v., i.p., s.c., respectively). **(d-f)** Western blot results, **(g-i)** corresponding quantification results (n=4, n=4 and n=6 in SAT, VFD, and liver respectively) in SAT, VFD, and liver, respectively of the mice with obesity accepted KT-NE treatment through different administration routes (i.v., i.p., s.c., respectively). Experiments were repeated 3 times independently, with similar results. Statistical significance was evaluated by a paired two-tailed t-test. All data was expressed as mean ± SD, ns=no significance.

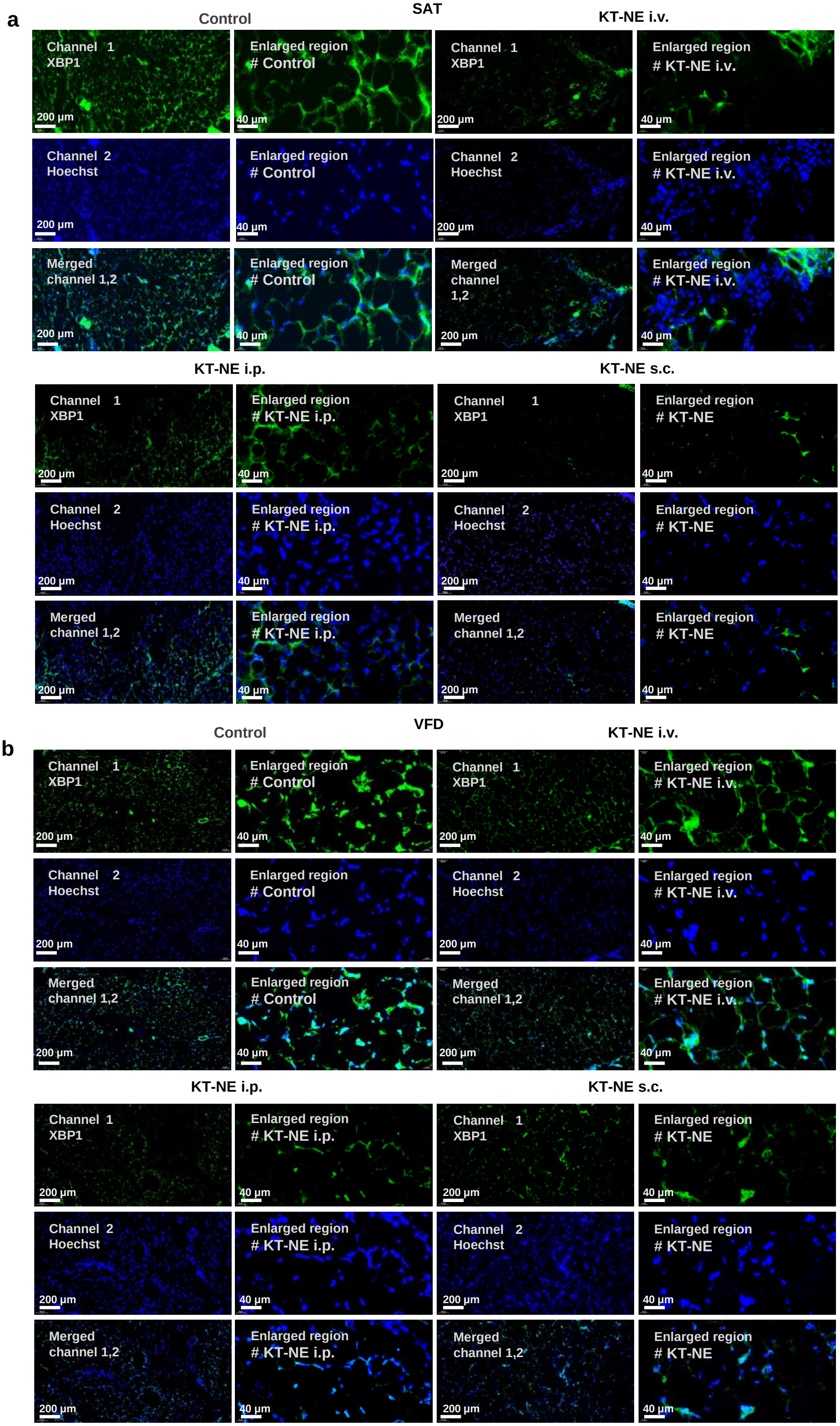

Supplementary Fig.12 The XBP1 expression in adipose tissues after treatment with KT-NE. (a) Representative immunofluorescence images depicting XBP1s expression in subcutaneous adipose tissue (SAT) and **(b)** visceral fat depots (VFD) respectively of the mice with obesity following treatment with KT-NE through various administration routes (i.v., i.p., s.c., respectively). Scale bar=200 µm, and scale bar=40 µm in the enlarged images.

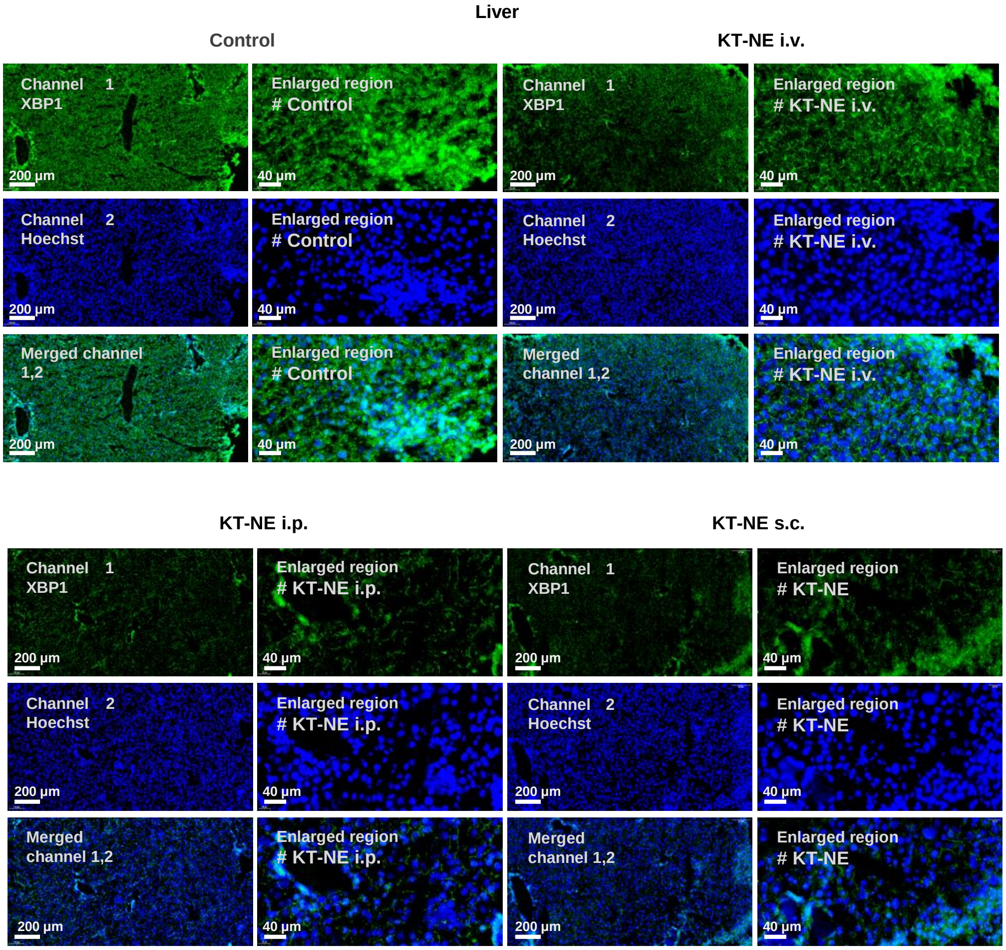

Supplementary Fig.13 The XBP1 expression in the liver after treated by KT-NE. Representative immunofluorescence images of XBP1s expression in the liver of the mice with obesity that received KT-NE treatment via different administration routes (i.v., i.p., s.c., respectively). Scale bar=200 µm, and scale bar=40 µm in the enlarged images.
