# Supplementary material for: A nanoemulsion targeting adipose hypertrophy and hyperplasia shows anti-obesity efficiency in female mice
Source: Nat Commun. 2024 Jan 2;15:72. doi: 10.1038/s41467-023-44416-3 (PMC10761889; doi:10.1038/s41467-023-44416-3)
Supplement: Supplementary file 2 — Reporting Summary [file 41467_2023_44416_MOESM2_ESM.pdf]

nature portfolio

Corresponding author(s): Qingpo Li, Jian You, Lihua Luo

Last updated by author(s): Dec 8, 2023

Reporting Summary

Nature Portfolio wishes to improve the reproducibility of the work that we publish. This form provides structure for consistency and transparency in reporting. For further information on Nature Portfolio policies, see our Editorial Policies and the Editorial Policy Checklist.

Statistics

For all statistical analyses, confirm that the following items are present in the figure legend, table legend, main text, or Methods section.

n/a	Confirmed	
☐	☒	The exact sample size (n) for each experimental group/condition, given as a discrete number and unit of measurement
☐	☒	A statement on whether measurements were taken from distinct samples or whether the same sample was measured repeatedly
☐	☒	The statistical test(s) used AND whether they are one- or two-sided<br>*Only common tests should be described solely by name; describe more complex techniques in the Methods section.*
☒	☐	A description of all covariates tested
☒	☐	A description of any assumptions or corrections, such as tests of normality and adjustment for multiple comparisons
☐	☒	A full description of the statistical parameters including central tendency (e.g. means) or other basic estimates (e.g. regression coefficient) AND variation (e.g. standard deviation) or associated estimates of uncertainty (e.g. confidence intervals)
☐	☒	For null hypothesis testing, the test statistic (e.g. F, t, r) with confidence intervals, effect sizes, degrees of freedom and P value noted<br>*Give P values as exact values whenever suitable.*
☒	☐	For Bayesian analysis, information on the choice of priors and Markov chain Monte Carlo settings
☒	☐	For hierarchical and complex designs, identification of the appropriate level for tests and full reporting of outcomes
☒	☐	Estimates of effect sizes (e.g. Cohen's d, Pearson's r), indicating how they were calculated

Our web collection on statistics for biologists contains articles on many of the points above.

Software and code

Policy information about availability of computer code

Data collection	MILabs-Acquision-12.26 (micro CT), Thermo Scientific Varioskan Flash 5250040, ECLIPSE Ti C-HGFI Nikon; Biospace Optima
Data analysis	GraphPad Prism 8.4 (statistical analysis of the majority of in vitro and in vivo experiments). Image J (quantification of fluorescence intensity and western blot). IMALYTICS Preclinical 2.1.8.9 (CT images).

For manuscripts utilizing custom algorithms or software that are central to the research but not yet described in published literature, software must be made available to editors and reviewers. We strongly encourage code deposition in a community repository (e.g. GitHub). See the Nature Portfolio guidelines for submitting code & software for further information.

Data

Policy information about availability of data

All manuscripts must include a data availability statement. This statement should provide the following information, where applicable:

- Accession codes, unique identifiers, or web links for publicly available datasets
- A description of any restrictions on data availability
- For clinical datasets or third party data, please ensure that the statement adheres to our policy

All data supporting the findings of the paper are available in the Supplementary Information file and in the Source data file. Source data are provided with this paper.

Research involving human participants, their data, or biological material

Policy information about studies with human participants or human data. See also policy information about sex, gender (identity/presentation), and sexual orientation and race, ethnicity and racism.

Reporting on sex and gender	The study did not involve human research participants.
Reporting on race, ethnicity, or other socially relevant groupings	The study did not involve human research participants.
Population characteristics	The study did not involve human research participants.
Recruitment	The study did not involve human research participants.
Ethics oversight	The study did not involve human research participants.

Note that full information on the approval of the study protocol must also be provided in the manuscript.

Field-specific reporting

Please select the one below that is the best fit for your research. If you are not sure, read the appropriate sections before making your selection.

☒ Life sciences ☐ Behavioural & social sciences ☐ Ecological, evolutionary & environmental sciences

For a reference copy of the document with all sections, see nature.com/documents/nr-reporting-summary-flat.pdf

Life sciences study design

All studies must disclose on these points even when the disclosure is negative.

Sample size	Sample sizes are clearly reported in the Figure Legend. The animal studies were performed on different models (n ≥ 5 biological replicates for each group). No statistical methods were used to pre-determine sample sizes.
Data exclusions	No data were excluded from this research.
Replication	Data presented were repeated for at least three times (biological replicates) and the successful attempts at replication were confirmed.
Randomization	Animal groups were randomized by body weight. In the other experiments, samples were randomly assigned to experimental groups.
Blinding	Experimental groups animals were randomly allocated to each of the experimental groups

Reporting for specific materials, systems and methods

We require information from authors about some types of materials, experimental systems and methods used in many studies. Here, indicate whether each material, system or method listed is relevant to your study. If you are not sure if a list item applies to your research, read the appropriate section before selecting a response.

Materials & experimental systems

n/a	Involved in the study	
☐	☒	Antibodies
☐	☒	Eukaryotic cell lines
☒	☐	Palaeontology and archaeology
☐	☒	Animals and other organisms
☒	☐	Clinical data
☒	☐	Dual use research of concern
☒	☐	Plants

Methods

n/a	Involved in the study	
☒	☐	ChIP-seq
☒	☐	Flow cytometry
☒	☐	MRI-based neuroimaging

Antibodies

Antibodies used	anti-mouse XBP1 rabbit polyclonal antibody (proteintech, Cat No: 10624-2-AP), anti-mouse FASN polyclonal antibody (Proteintech, Cat No: 10624-2-AP), anti-mouse β-actin recombinant antibody (Proteintech, 81115-1-RR),HRP-anti-rabbit IgG (H+L) (Beyotime, A0208)

Validation	All antibodies were verified by the supplier and each lot has been quality tested. All validation statements are available on the antibody websites, respectively.<br>anti-mouse XBP1 rabbit polyclonal antibody: https://www.abcam.cn/products/primary-antibodies/xbp1-antibody-epr22004-ab220783.html<br>anti-mouse FASN polyclonal antibody: http://www.ptgcn.com/products/FASN-Antibody-10624-2-AP.htm<br>anti-mouse β-actin recombinant antibody: https://www.ptgcn.com/products/beta-actin-Antibody-81115-1-RR.htm<br>HRP-anti-rabbit IgG (H+L): https://m.beyotime.com/product/A0208.htm

Eukaryotic cell lines

Policy information about cell lines and Sex and Gender in Research

Cell line source(s)	The preadipocyte cell line 3T3-L1 was purchased from American Type Culture Collection (ATCC®, CL-173™), primary umbilical vein endothelial cells (HUVEC) (ATCC®, PCS-100–010™), human embryonic kidney cells HEK-293 (ATCC®, CRL-1573™), immortalized keratinocytes HACAT, normal liver cells L-O2 (BeNa Culture Collection, Beijing, China, BNCC360120) and as well as mouse C2C12 myoblasts cells (BeNa Culture Collection, Beijing, China, BNCC338573).
Authentication	Cell lines are tested and authenticated by the Institute of Biochemistry and Cell Biology in a systematic process including morphology assessment, karyotyping, and PCR-based approaches. Following receipt from the Institute of Biochemistry and Cell Biology, the identity of the cell lines were confirmed through assessment of cellular morphology under microscope.
Mycoplasma contamination	Mycobacterium negative; bacterium and yeast negative; HIV-1, hepatitis B and hepatitis C negative.
Commonly misidentified lines<br>(See ICLAC register)	No commonly misidentified cell lines were used.

Animals and other research organisms

Policy information about studies involving animals; ARRIVE guidelines recommended for reporting animal research, and Sex and Gender in Research

Laboratory animals	Female 8-10 week-old C57BL/6 mice (Slaccas Experimental Animal Co., Ltd. Shanghai, China) were canonically bred and maintained under pathogen-free conditions. And female 8-10 week-old C57BL/6 mice were fed with 4-week-high-fat diets (FBSH Biotechnology Co.,Ltd., D12492, containing 60% fat, 4.75 Kcal/g) to build mice model with obesity. Mice were housed in a standard laboratory environment at 22~25°C, humidity maintained at 30% ~70%, in the 12h light/dark cycle (9 am-9pm lights on, and 9 pm-9 am lights off) and were allocated to experimental groups randomly.
Wild animals	The study did not involve wild animals.
Reporting on sex	Only female animals were used in this study.
Field-collected samples	The study did not involve field-collected samples.
Ethics oversight	All experiments were carried out according to the requirements of the Zhejiang University Animal Study Committee (approval no. 23044). All in vivo experiments were performed in compliance with the requirements of the Zhejiang University Animal Study Committee for the care and use of laboratory animals in research.

Note that full information on the approval of the study protocol must also be provided in the manuscript.
